# The human experience of social transformation: Insights from comparative archaeology

**DOI:** 10.1371/journal.pone.0208060

**Published:** 2018-11-29

**Authors:** Michelle Hegmon, Matthew A. Peeples, on behalf of the LTVTP-NABO collaboration

**Affiliations:** 1 School of Human Evolution and Social Change, Arizona State University, Tempe, Arizona, United States of America; 2 Center for Archaeology and Society, Arizona State University, Tempe, Arizona, United States of America; University at Buffalo - The State University of New York, UNITED STATES

## Abstract

Archaeologists and other scholars have long studied the causes of collapse and other major social transformations and debated how they can be understood. This article instead focuses on the human experience of living through those transformations, analyzing 18 transformation cases from the US Southwest and the North Atlantic. The transformations, including changes in human securities, were coded based on expert knowledge and data analyzed using Qualitative Comparative Analysis techniques. Results point to the following conclusions: Major transformations, including collapses, generally have a strong and negative impact on human security; flexible strategies that facilitate smaller scale changes may ameliorate those difficulties. Community security is strongly implicated in these changes; strong community security may minimize other negative changes. The relationships among the variables are complex and multi-causal; while social transformation may lead to declines in human securities, declining conditions of life can also push people to transform their societies in negative ways. Results show that some societies are better able to deal with difficulties than others. One important policy implication is that community security and local conditions can be instrumental both in helping people to cope with difficulties and in staving off some of those difficulties. A multi-scalar approach is essential as we face the increasing problems of climate change in the decades ahead.

## Introduction

Bad news is also often big news. Of all the subjects investigated by archaeologists, few garner as much interest from the general public as the end of civilizations. Former President Obama is reported to be a fan of Jared Diamond’s *Collapse* and discussed climate policies in response to a question about the collapse of civilizations [1,2]. Columnist Nicholas Kristof recently used Easter Island as an example in his column on the parable of self-destruction and climate change [3]. The *New York Times* reported on research showing that even careful preparation and planning could not stop what they called the “collapse” of the Egyptian empire in the face of severe drought [4].

In spite of, or perhaps because of this public attention, archaeologists now often eschew the word “collapse” or define it cautiously. There have been a host of criticisms of Jared Diamond’s *Collapse*, including an entire volume focused on reevaluating the cases [5]. Joseph Tainter’s seminal work defined collapse as a loss of complexity [6,7], and he has since argued that changes in complexity, including collapses, are part of “a normal evolutionary process” [8]. Recent work emphasizes studying collapse in tandem with resilience, revitalization, and transformation [9]. In other cases, archaeologists are finding demographic continuity across the end of a cultural sequence and thus arguing these changes should be understood as “reorganizations” or “transformations” rather than collapses [10,11]. Similarly, it is now understood that indigenous people often maintain connections to places where their ancestors lived and that these sites were not “abandoned” [12,13]. Finally, some emphasize the constructive lessons we can learn from the past, including difficult times in the past [14,15].

We largely agree with, and indeed have participated in, these scholarly calls for caution [16,17]. At the same time, we are concerned that the pendulum is swinging too far towards normalizing these major events. In large part, this is a matter of scale. In the longue durée of centuries and millennia, the rise and fall of civilizations is part of the normal course of history. But from the perspective of people who live through them, these events have life-changing consequences. Perhaps this is why today’s news is so full of stories about the ends of civilizations, because we fear difficult times ahead.

The present research reconciles some of these perspectives, in two respects. First, we consider a range of *social transformations*, defined as lasting and major changes in settlement, economy, and/or socio-political organization such that people’s life experiences before and after the transformation are substantially different. This definition subsumes collapses but also includes other kinds of changes [9]. Social transformations can be rapid, occurring within the life span of an individual, or they can be more gradual and develop over the course of several generations. Social transformations are important both because they involve changes in the workings of society as a whole, and because they affect the lived experiences of the people in those societies, our second point. Most work on transformations has focused on institutions and society-wide process; one recent exception is a study of household adaptation [18]. Here, while we consider these large-scale processes, we add a focus specifically on the *human experience* of these transformations, building on archaeological approaches that assess the conditions or quality of life in the past [19,20]. Importantly, the process is far from unilinear—it is not just a matter of change at a large scale affecting people at a smaller scale. On the contrary, people’s lived experiences also affect or even cause transformations, the subject of considerable research in the literature on social movements [21,22] and resistance [23,24].

Our research assumes that social transformations, even those that are not true collapses, are often difficult for the people who experience them, and our general goal is to document and understand those difficulties: What kinds of changes are the most difficult and for whom? What factors might ameliorate those difficulties? To this end, our research draws on the long time span of the archaeological record to develop a widely applicable understanding of social transformations, an understanding that considers both society-wide processes and people’s lived experiences [25]. In past work, we and our collaborators focused mostly on single instances or areas [19,26,27,17,28,29]. Here, we expand this view, comparing transformations in eight archaeological and historical sequences from two very different areas of the world, the US Southwest and the North Atlantic. Specifically, we analyze a series of variables for each transformation and the period that immediately preceded it to assess both the nature of the transformation and the way it affected peoples’ lives. The specific goals are:

1. Develop a method for comparing different kinds of transformations and the human experience of those transformations in different kinds of settings based on different kinds of data.
2. Explore ways of classifying transformations based on their characteristics and thus evaluate the utility of a typology of transformations.
3. Explore the relationship between social transformations and people’s lived experiences. Are some changes easier or more difficult than others? How might people’s lived experience affect transformations and other large-scale social processes?
4. Consider the implications of these results for today’s world.

## Materials and methods

### Approaches to social transformations

A brief review of approaches to social transformations sets the stage for our analytical focus and approach. Past work by other authors and ourselves provide the basis for (1) the variables selected and used in this analysis, (2) methods of classifying transformations, and (3) means of gathering necessary data.

Archaeologists, historians, and others have long studied severe transformations, including collapses [6,9,29–32]. Tainter, who emphasizes the loss and change of institutions, defines socio-political collapse as “a rapid simplification, the loss of an established level of social, political or economic complexity” [33]. Turner and Sabloff argue that the term collapse is appropriate to describe the Maya Terminal Classic Period because “the Central Maya Lowlands and its large infrastructure of cities, water systems, and managed landscapes, were essentially abandoned, with population declines approaching 90%, and it remained so for well over a millennium” [32]. In the remainder of this article, which considers a broad range of social transformations, we use the term “collapse” sparingly but still draw on the careful definitions and analyses of these authors. Specifically, the considerable body work points to the importance of institutional and demographic change and thus two variables that become key in our analyses, degree of *institutional breakdown* and *depopulation*.

Some recent work has focused on other kinds of transformations. Nelson and colleagues [34] and Torvinen and colleagues [35] introduced the concepts “continuity with change” and “transformative relocation” to describe major changes that do not fit neatly under the heading of collapse but still constituted dramatic and jarring changes at the scale of regions. Continuity with change describes situations in which there were major institutional changes but general continuity of the population and settlement. Transformative relocation refers to situations in which there were both changes in institutions and a major relocation or decline of the population though not to the point of collapse. Yet another category is “reorganization” in which people changed their institutions and moved to different kinds of settlements but stayed in a region [11,16]. This work on other kinds of transformations also points to the importance of institutional and demographic change.

These typologies of transformations provide useful structure, though some cases may not fit well into the limited number of categories. A different approach, which we have used previously, characterizes transformations in terms of a series of variables assessed with archaeological proxies [17]. For example, rather than classifying a transformation as either “collapse” or “transformative relocation,” our 2008 analyses considered the scale and degree of demographic displacement, assessed in terms of population size and evidence of emigration. The current article uses this follows this approach variable-based approach. Rather than defining types *a priori*, the present work begins with variables that are then used to group cases and explore the utility of a typology (Goal # 2).

Third, focus on the human scale in social transformations requires information on numerous processes and variables, ranging from food security and labor to violence and inequality. Some of these can be assessed with proxy measures–for example, using Gini coefficients of household size to assess inequality [36,37] or using skeletal data to develop indices of violent trauma [38]. However, other important processes cannot be measured in this way, and proxies are often not able to assess important nuance suggested by expert knowledge. Thus, the current work develops a means of systematically incorporating expert knowledge through qualitative comparative analysis.

### The human experience

The field of international development has long been concerned with measuring and comparing the conditions of life in different times and places, in part to assess the effectiveness of their development programs. As a result, they have created host of methods for making such measurements. Some focus on specific variables such as food insecurity [39]. Others develop multiple measures to assess the nature of poverty and inequality [40–42], the overall quality of life [43] or level of human security [44–46].

In recent years, archaeologists have begun to apply these measures to study the conditions of life. Arponen and colleagues [47] applied the capability approach in their analysis of inequality. Smith [20] uses quality of life concepts to study the impact of the Aztec expansion on several outlying communities. Hegmon developed the Archaeology of the Human Experience [19], which employed various methods including a system for operationalizing dimensions of human security; these dimensions were originally developed by the United Nations Development Programme [46] to bring focus on the human condition at a time when governments were emphasizing national security. The UNDP used seven dimensions–economic, food, health, environmental, personal, community, and political security–many of which can be assessed with archaeological data [48]. Some archaeological applications utilize all seven [49] while others have taken one or a few and considered their multiple components, such as Logan’s work which assessed the food security in terms of availability, access, use, and preference [50,51]. The research reported here considers all seven dimensions.

### Comparative analysis in archaeology

Archaeology is a strongly comparative discipline, and recent years have seen increasing interest in the methods and theory of comparative analysis [52–55]. Increasingly, comparative analysis is often used in studies that draw on archaeological data to address pressing issues in the contemporary world [25,29,56–58]. The archaeological cases are sometimes viewed as long-term “natural experiments” [59–61], which provide a valuable diachronic perspective [62]. Furthermore, a comparative analysis of these long-term experiments makes it possible to assess the relationship between possible causes and effects and thus evaluate theories about the causes of social change and even collapse. For example, Peregrine [58] analyzed 33 archaeologically known societies that had faced climate-related disasters and that had more and less participatory political structures; his comparisons allowed him to conclude that political participation provides resilience to climate-related disasters.

Comparative work in archaeology has taken a variety of forms and has been conducted at many scales [55]. Several of the most influential works rely on detailed descriptions of a small number of cases set side-by-side to explore common or divergent trajectories of some phenomenon of interest, such as social complexity or early state formation, with comparisons made largely through narrative discussion [63,64]. Other studies focus on larger numbers of cases to make data-centered comparisons; they use statistical methods to reveal systematic variation across contexts with the goal of identifying potential causal relationships between variables and outcomes of interest. In some cases, this takes the form of analyses of huge standardized datasets with many cases and variables, sometimes drawing on resources like the Human Relations Area Files [53,54,58,65,66].

Most recent data-centered archaeological comparative research falls somewhere in the middle with moderate numbers of cases (on the order of 10–20) compared directly across a small number of dimensions of variation, with the goal of making both highly contextualized and broad comparative arguments. This middle-ground has been quite productive and archaeologists have developed many of their own methods for making such comparisons, often combining both archaeological and contemporary data [25,52,67–69]. This situation in archaeology mirrors that in the broader comparative social sciences in many ways, including the increasing importance of this middle-ground between small-n and large-n comparisons [70,71]. Here we draw on this broader body of work and explore the potential utility of recent approaches to Qualitative Comparative Analysis (sometimes called Configurational Comparative Methods) for archaeological comparisons.

### Qualitative comparative analysis

The analyses presented here are based on methods of qualitative comparative analysis (QCA) developed by Charles Ragin [70–72] as well as recent extensions of the QCA approach by Breiger [73]. Despite the word “qualitative,” QCA does use quantitative methods and thus does not fit the usual definition of qualitative research (e.g., http://journals.plos.org/plosone/s/submission-guidelines#loc-qualitative-research). QCA refers to a set of case-based comparative methods designed for making detailed and systematic comparisons among moderate numbers of cases (usually 10–40). QCA is focused on characterizing the interactions among those cases and variables, which may be either causal conditions or outcomes, to identify potential relationships of necessity (one condition necessitates another) and sufficiency (one condition is sufficient for causing another, but not necessary). QCA methods have rapidly gained popularity in a number of fields, in particular sociology and political science, and have been directed towards a wide variety of social and environmental phenomena and processes (see http://www.compass.org/ for a bibliography of more than 250 recent publications using QCA methods).

QCA approaches are based on set relationships. A set, in QCA parlance, is simply a grouping such that a particular case can be defined as either within or outside. As a simple example the numbers 2, 3, 5, 7, and 11 are all within the set of prime numbers and are also in the larger set of positive integers. Proponents of QCA approaches argue that set membership can be extended beyond simple definitions to explorations of causal relationships among cases and variables. Specifically, Ragin (1987) argues that many causal arguments are set-based at their core. For example, we might be interested in determining the degree to which groups that are within the set of "agriculturally dependent" societies are also within the set of "sedentary" societies. In this example, both variables (sedentism and agricultural dependence) can be treated as either potential causal conditions or outcomes.

As the previous example illustrates, membership in many of the sets we may be interested in exploring may vary by degree. To deal with such variation Ragin [70,72] and others [74,75] have extended traditional QCA methods to accommodate groupings based on "fuzzy sets." Fuzzy set QCA (fsQCA) allows for differentiation in set membership based on analyst- defined conditions that delineate full membership in a set, partial membership in a set, as well as full and partial non-membership. Researchers use empirical knowledge of cases to define *qualitative anchors* delineating degrees of membership in such fuzzy sets.

As there is often no simple way to define a metric for measuring degrees of membership in sets, researchers often must rely on pre-defined coding schemes characterizing full membership (usually coded as 1) and non-membership (usually coded as 0) of cases in sets as well as specific points of ambiguous membership (numbers between 0 and 1). In the analyses presented in this paper, we use a common four-part scale; 1—full membership in a set, 0.75—more in a set than out, 0.25—more out of a set than in, and 0—fully out of set. Note that this is not the same as a rank order classification, as we explicitly define qualitative anchors that denote full or non-membership in the set. In other words, once a case is defined as either in or out of a set, variation above or below these qualitative anchors is seen as less important and all cases belonging to a particular set are treated as equal members of that set. To return to our earlier example, societies that engage in small-scale dry farming and industrial farming are both full members of the set of agricultural societies, and variation in practices between them are deemed less important than their membership in that set. Set membership can often be assessed in terms of relatively simple yes or no questions. In the analyses presented here, our cases are specific periods of social transformation as defined above. One of the sets we are interested in assessing is whether or not each transformation was marked by immigration (designated as IMG). To place each case along this four-part coding scheme of set membership, we devise a carefully worded question and the specific criteria for each level of set membership.

**In-migration (IMG)**

***Was the transformation associated with evidence for the immigration of new individuals or groups into the region?***

**yes (1)—**Evidence for the establishment of new communities or substantial segments of existing communities by immigrants from outside the region.

**more yes than no (.75)—**Evidence for immigrants joining existing communities (or the establishment of new local/immigrant communities).

**more no than yes (.25)**—Limited evidence for immigrants in the region with limited or uncertain impacts.

**No (0)—**Little to no evidence for new immigrants in the region.

In this research, we use this basic four-part coding scheme and the approach described here to code all our cases for variables described in more detail below. Importantly, as this brief example illustrates, this method of fuzzy set coding relies on expert knowledge of cases, supported by specific examples and case-based knowledge, for exploring the process or phenomenon in question. The approach is particularly useful in comparisons of widely differing cases where it is often difficult or impossible to find reasonable proxies that work for all of the cases.

Traditional methods for QCA and fsQCA often rely on analyses of what are called truth tables; these are two-way tables quantifying the membership of cases in sets in relation to causal conditions and outcomes used to identify and test conditions of necessity and sufficiency among variables. Breiger [73] has recently published an extension of the fsQCA approach based on correspondence analysis (CA). This CA approach has proven to be a powerful method of exploratory analysis focused on developing hypotheses about the potential relationships between variables and cases. CA already has a long history in archaeology, in particular in work focused on chronological seriation [76–80]. CA provides a method of displaying a large proportion of the variation and complex inter-relationships present within a two-way table of cases and variables in low-dimensional space. In many ways, CA is a natural fit for QCA data in that this method of ordination allows for the simultaneous visualization of cases and variables. As the focus of the current study is hypothesis building and assessing relationships among cases and key variables, we rely on this CA approach to QCA data.

### The LTVTP-NABO collaboration and our database

The data for this study are derived from multi-layered collaborative research that provides information on social transformations in two very different areas. The Long- Term Vulnerability and Transformation Project (LTVTP) focuses on cases in the arid and semi-arid US Southwest and northern Mexico. The North Atlantic Biocultural Organization (http://www.nabohome.org/) focuses on the subarctic and arctic North Atlantic. Both projects have separately developed comparative perspectives across their cases [17,35,81,82]. More recently, the two have developed a cross-area comparison focused on understanding human vulnerability to climate change [25,83,84]. The study of social transformations is part of this work both because transformations may affect vulnerabilities and because they sometimes result, at least in part, from climate change.

#### Transformations

The LTVTP-NABO group as a whole (recognized in the acknowledgements) defined a series of social transformations that are the basis of the research described in this paper. The transformations follow the definition set forth above: “Lasting and major change in settlement, economy, and/or socio-political organization such that people’s life experiences before and after the transformation would be substantially different.” They are recognized by major changes in the archaeological (and sometimes historical record) such as changes in settlement, organization, or population. The transformations (ten from the Southwest, eight from the North Atlantic) are listed in Table 1 and described in more detail in the Appendix, available as S1 Table.

**Table 1:** The transformations cases analyzed in this study.

Transformation Case and Code	Date CE	Periods
**North Atlantic**		
Greenland (GE1)	980–1000	*Landnám*
Establish unique settlement-subsistence system: pastoralism and seals for domestic use, walrus for market and trade.		
Greenland (GE2)	1250–1300	Recession
Beginning of decline in pastoralism and increased dependence on seal.		
Greenland (GE3)	1400-1450/70	End of Norse Settlements
Contacts with Europe lost, remaining population dies or leaves. End of Norse occupation.		
Iceland (I1)	870–890	*Norse Landnám*
Rapid settlement.		
Iceland (I2)	950–1000	Consolidation
System of law and order is established. Formation of Icelandic identity. Christianization.		
Iceland (I3)	1250–1300	Economic and Political Threshold
Economic changes and administrative reorganization.		
Faroes (F1)	800–850	*Norse Landnám*
Norse/Celtic settlement, establish domestic economies.		
Faroes (F2)	1250–1300	Sociopolitical reorganization.
Sociopolitical reorganization including changes in land use and settlement institutionalized with the Sheep Letter.		
**Southwest**		
Zuni (Z1)	1250–1290	Pueblo III–Pueblo IV
Movement from dispersed settlements to formal, plaza-centered nucleated towns through region.		
Zuni (Z2)	1375–1400	Pueblo IV–Protohistoric
In-migration and movement of entire population to a few towns in the Zuni river valley.		
Salinas (S1)	1275–1325	Jacal–Masonry
Consolidation of loosely clustered jacal communities into single, possibly defensible, masonry structures.		
Salinas (S2)	1400–1425	Late Pueblo
Consolidation of communities into a small number of large pueblos in new (off mesa) locations.		
Hohokam (H1)	1070–1100	Sedentary–Classic
End of the regional system, Balkanization. Concentration of population in the Phoenix Basin.		
Hohokam (H2)	1375–1450	End of Classic
Slow decline at end of Classic, end of massive irrigation system, depopulation.		
Mimbres (M1)	950–1000	Pithouse–Classic
Deliberate burning of great kivas, shift to above ground pueblos with smaller ritual structures, elaborate pottery.		
Mimbres (M2)	1130–1150+	Classic–Reorganization
Depopulation of large villages, some move to smaller hamlets; end of pottery tradition, more outside connections.		
Mesa Verde (MV1)	880–920	End of Pueblo I
Simplification and then depopulation of large Pueblo I villages; big population decline across region.		
Mesa Verde (MV2)	1240–1290	Pueblo III depopulation
Period of violence and probably subsistence stress culminating in large scale abandonment of region.		

#### Variables

Our research goals include exploring ways of classifying the transformations as well as understanding their effects. To this end, the analysis is based on three sets of variables, listed in Table 2. Each variable was assessed for each case, following QCA procedures. Two *key characteristic* variables assess the issues emphasized in the literature on collapse and transformation discussed above, these are, institutional breakdown (INST) and depopulation (DPOP).

**Table 2:** Variables assessed for each transformation.

**Key Variables**
**INST—**institutional breakdown
Was the transformation characterized by a breakdown of institutions that would have been part of societal complexity?
**DPOP–**depopulation
Was the transformation characterized by regional scale depopulation or a high degree of population loss?
**Nature of Change Variables**
**IMG—**in-migration
Was the transformation associated with evidence for the immigration of new individuals or groups into the region?
**DIV**–material culture diversity
Was the transformation associated with changes in material cultural diversity (more or less) potentially marking new or shifting networks of regional scale interaction and identity?
**OUT**—outside influence
Was the transformation associated with increasing outside influence in the study area?
**HORG**–household organization
Was the transformation associated with substantial changes in household scale social organization?
**CORG**–community organization
Was the transformation associated with substantial changes in community scale social organization?
**TRD–**interregional trade
Was there a change in interregional trade that would have made it more difficult to get important goods?
**Human Security Variables**
**FDShort**–food security
Is there evidence of a decline in the availability of food, for at least some sector of society?
**ENVSEC**—environmental security
Was there a decline in environmental security?
**TRD–**economic security, trade
Was there a change in interregional trade that would have made it more difficult to get important goods?
**PROD–**economic security, production
Was there a change that increasingly alienated people from their means of production (e.g., land, boats, tools, irrigation networks)?
**COMM–**community security
Did communities disintegrate or disappear?
**VIO**–personal security
Was there an increase in violence?
**HEAL**–health security
Was there a decrease in health from causes other than nutritional deficiencies (e.g., epidemics, the plague)?
**POWD–**power differences
Was there an increase in power differentials, such that some people increasingly have power over others and the “others’” experience a loss of autonomy?

Six *nature of change* variables are derived from our collaboration’s many discussions of the transformations, and they describe factors that were important in at least some cases and that could be assessed in most. These six variables, for example, changes in household organization (HORG) or and material culture diversity (DIV), are designed to assess factors that are generally neutral, not necessarily desirable or problematic.

Seven *human security* variables assess the transformations in terms of the UNDP’s dimensions of human security. In contrast to the nature of change variables, these do concern issues that would have affected the quality of life, such as a decrease in food security (FDShort) or personal security (VIO). Of the seven human security variables, six are derived directly from the UNDP approach. The seventh, the UNDP dimension “political security” concerns basic human rights and is concerned with issues such as political repression and detention. These issues are relevant in some of our cases, but cross-case evidence is sparse. Thus, we instead focus specifically on issues of inequality and power differences. While a certain degree of inequality was the norm in most or all of the societies we are studying [26], we consider an increase in the degree of inequality or power differences (POWD) to be one of the human security variables, since it is likely that such decreases would have led to a decline in lower ranking people’s conditions of life.

Change in community organization (CORG) is one of the nature of change variables and decrease in community security (COMM) is one of the human security variables. Although the two are related, they concern different kinds of issues, the latter focusing specifically on the disintegration or disappearance of communities. For example, the Norse settlement of Iceland and the Faroes (**I1** and **F1**) involved the establishment of new communities and thus major changes (CORG = 1 and .75) that would have strengthened community security (COMM = 0).

Each transformation was coded for these variables, drawing on expert knowledge supported by a variety of published work and other analyses. Codes were assigned based on a series of questions, as in the immigration example described above. The questions were designed to focus specifically on change through the course of transformation, with no change assigned a score of 0, and major change a score of 1. For the human security variables, a higher score indicates a decline in security and a worsening of conditions. Inter-case comparisons were based on the directions of change observed within each sequence, not on absolute levels. For example, the analysis did not attempt to determine if there was greater food security in Zuni than in Greenland; rather, it compared the direction of change in food security through the several Zuni and Greenland transformations. The questions used to assess each variable, resultant codes for each case, and justification for these codes are detailed in the Appendix (S1 Table). Examples of the coding are provided in Supplement 2 (S1 Text). Table 3 summarizes the results, which are the basis of the following analysis.

**Table 3:** Coded QCA variables for all cases.

		Key Variables		Nature of Change Variables						Human Security Variables						
Transformation	Dates (A.D.)	*INST*	*DPOP*	*IMG*	*DIV*	*OUT*	*HORG*	*CORG*	*TRD*	*FDshort*	*ENVSEC*	*PROD*	*COMM*	*VIO*	*HEAL*	*POWD*
**North Atlantic Cases**																
GE1	980–1000	0	0	1	0	0	0	.25	.75	0	0	0	0	.25	0	0
GE2	1250–1310	0	.25	0	0	.25	.25	.25	.25	.75	.25	.25	0	0	0	.25
GE3	1400-1450/70	1	1	.25	0	0	.25	0	1	1	.25	0	1	0	0	0
I1	870–890	0	0	1	0	.25	.75	1	.75	0	.75	0	0	.25	0	.75
I2	950–1000	0	.25	.25	1	.75	.25	.25	0	0	1	0	.25	0	0	.25
I3	1250–1300	0	0	0	.25	.25	.25	0	0	.75	1	0	.25	0	0	.25
F1	800–850	0	0	1	0	.25	0	.75	.75	0	0	0	0	.25	0	0
F2	1250–1300	0	0	0	.25	1	.25	0	0	0	0	.75	0	0	0	.75
**Southwestern Cases**																
Z1	1250–1290	0	0	.25	0	0	.25	1	0	0	0	0	.75	.75	.25	0
Z2	1350–1400	0	0	.75	.75	.25	.25	.75	0	0	0	0	.75	0	.25	0
S1	1275–1325	0	0	0	.25	.25	0	1	0	.25	0	.25	0	1	0	.25
S2	1400–1425	0	0	0	.75	.75	0	1	0	.75	.25	0	.25	0	.25	.25
H1	1070–1100	.75	.25	.25	.25	0	.75	.75	1	.25	.25	.75	.75	.25	.25	1
H2	1375–1450	1	1	.25	0	.25	1	1	1	1	.75	1	1	0	.25	1
M1	900–1000	.25	0	.75	0	0	.75	.75	.75	0	0	.25	.25	0	.25	.25
M2	1130–1150+	1	.75	.25	1	1	1	1	0	.25	.75	0	1	0	0	0
MV1	880–920	.75	.75	.25	0	0	.25	.25	.25	.25	.25	.25	.75	.75	0	.25
MV2	1240–1290	1	1	.75	.75	0	0.25	1	.75	.75	.75	.75	1	1	.25	.75

### Approach to correspondence analysis

The approach to QCA employed here involves subjecting the variable codes presented above to correspondence analysis (CA) to evaluate interrelationships among cases and variables visually and to develop potential hypotheses regarding the nature and severity of transformations. As described briefly above, CA is useful specifically because it allows for the simultaneous visualization of both cases and variables in the same low-dimensional space. Although the spatial relationships among cases and among variables in a CA plot are each independently meaningful (in other words cases or variables in similar portions of a CA plot are marked by similar relationships), the distances between cases and variables are not strictly defined [85]. To address this issue, this work follows the approach to scaling our CA results outlined by Breiger [73]. Specifically, we conduct CA using both the original variable codes, designated by the capitalized variable code designations shown in Table 3, as well as the inverse of those variable codes (1—variable code), designated by the same code in all lower-case letters. Case and variable CA scores are then scaled such that each case point falls at the weighted mean of the CA scores of the variables. Standard (principal coordinates) scaling is used for the row coordinates and barycentric scaling for the column coordinates [73]. For every variable, a line between the original variable (e.g., IMG) and its inverse (e.g., img) crosses directly through the origin of the CA plot (the 0,0 point). This approach facilities meaningful interpretations of the spatial relationships between variables and cases. Cases are pulled in the direction of the variables that constitute them. Similarly, the distances between cases are defined such that cases that are close together in the CA space are marked by similar relationships to the variables in question.

## Analyses and results

### Finding structure

**Fig 1:**
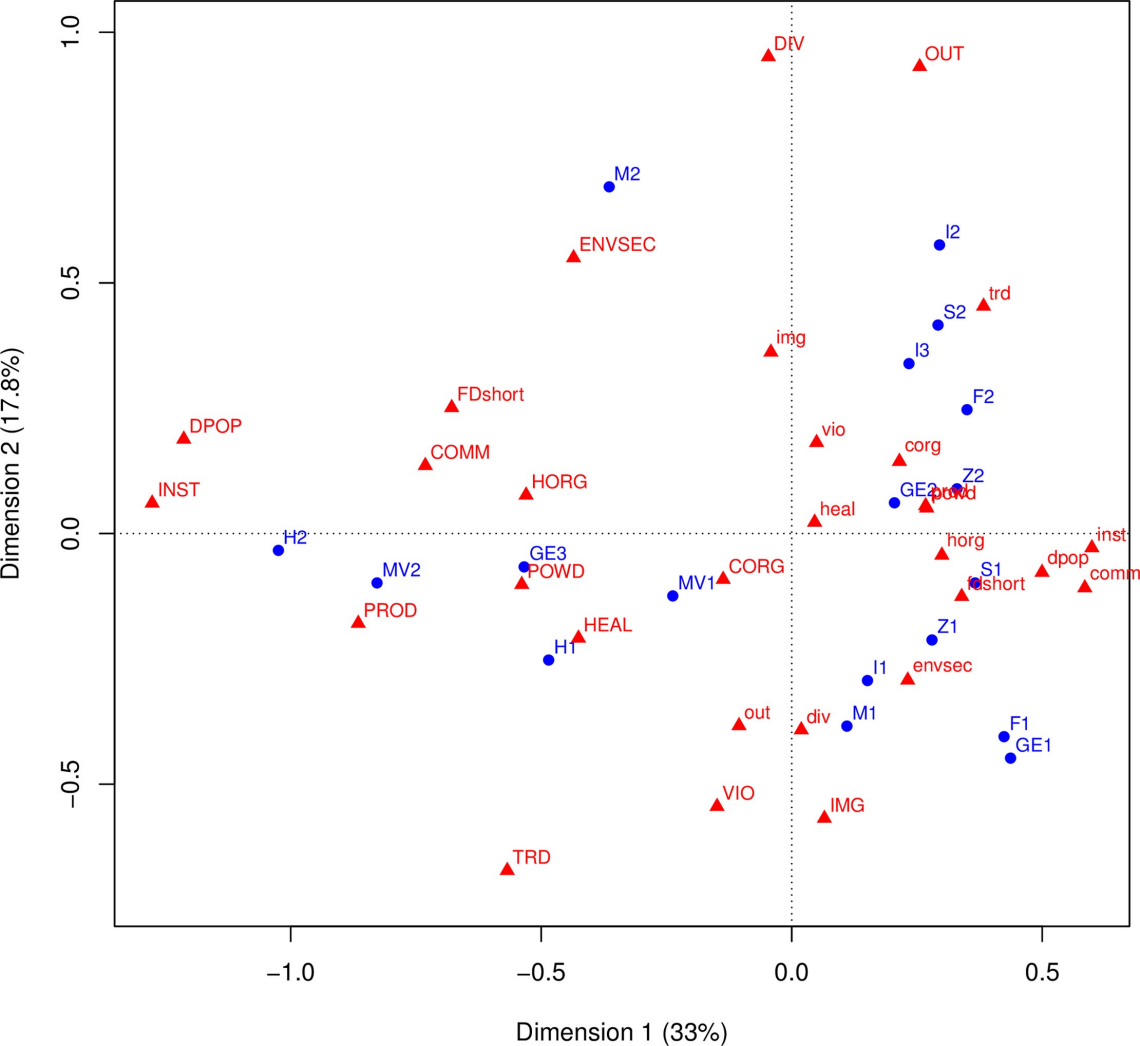
Correspondence analysis of all variables and transformation cases. Transformation cases, listed in Table 1, are in blue. Variables (in CAPS) and their inverse (lower case) are in red here and listed in Table 2.

To explore the overall distribution of the variables and cases, a single correspondence analysis (CA) is used to consider them all together in a plot, shown in Fig 1, with cases in blue and variables and their inverse in red (inverse indicated by lower case letters). This kitchen sink approach does not separate the cases in any easily interpretable way, but it shows some general patterns and demonstrates the approach. The cases from the two areas are interspersed, suggesting both experienced the same general processes. Changes in community organization are often accompanied by changes in household organization. For example, in the Mesa Verde 2 (**MV2**) transformation, aggregation into large defensible villages involved changes in both community and household organization; similarly, in the Hohokam 1 (**H1**) transformation, settlement consolidation was manifested at both scales. Thus, COMM (community organization) and HORG (household organization) plot close to one another. Three transformations that represent the termination of a sequence (Hohokam 2 [**H2**] Greenland 3 [**GE3**], and Mesa Verde 2 [**MV2**]) also plot near one another; all three are characterized by major institutional breakdown (INST = 1) and depopulation (DPOP = 1). The R code for this and all other analyses and figures presented here is available on line, as detailed in the Data Availability Statement.

The (Fig 1) plot also shows an association between the two key variables institutional breakdown (INST) and depopulation (DPOP). They plot near to one another (at the far left), though they not isomorphic. The most dramatic transformations, such as the end of the Norse occupation of Greenland (**GE3**), the end of the Hohokam sequence (**H2**), and the depopulation of the Mesa Verde region (**MV2**) all have almost total population decline (DPOP = 1) and major institutional breakdown (INST = 1). However, the end of the Mimbres Classic period (**M2**) is characterized by major institutional change (INST = 1) but some population continuity (DPOP = 0.75). In another instance, the Sedentary-Classic shift in the Hohokam region (**H1**) is characterized by major institutional changes (INST = 0.75) but population continuity (DPOP = 0.25).

The complex relationship between institutional breakdown and depopulation is shown in Table 4. In most cases the two are strongly associated, but there are exceptions, discussed above. The oft-discussed collapse of the Roman Empire [6] would be placed in the lower left quadrant because it involved a breakdown of institutions, the Roman system, but not a major population change across the formerly controlled area. The collapse of the Classic Maya Lowlands would be placed in the lower right quadrant because it exhibited both a breakdown of institutions and a substantial loss of population [32].

**Table 4:** Transformation cases sorted by the two key variables depopulation and institutional breakdown.

institutional breakdown score	depopulation score			
	0	0.25	0.75	1
**0**	GE1 I1 I3 F1F2 Z1 Z2 S1 S2	GE2 I2		
**0.25**	M1		H1	
**0.75**			MV1	
**1**			M2	GE3 H2MV2

Overall, these results show that the key variables institutional breakdown and depopulation co-vary strongly and cases with high scores on the key variables group together, indicating that they are similar according to other variables as well. However, cases of social transformations that involved other kinds of changes but lower scores on these key variables vary widely and do not appear to fit neatly into any typology. Thus, in the following section, we consider the two key variables separately, in relation to the other sets of variables describing the nature of change and changes in human security.

### Institutional breakdown and depopulation

Drawing on the large literature on collapse, our analyses defined two key variables that are part of the most dramatic transformations, institutional breakdown (INST) and depopulation (DPOP). The broad analysis described above revealed that high scores on the two co-vary, but lowers scores do not, and cases with lower scores on them are quite variable. In this section we consider each of the two key variables separately in relation to the sets of variables that describe the nature of change and changes in human securities. These analyses pursue Goal #2, the classification of transformations, and they also address Goal #3, exploring the relationships between transformations and people’s lived experiences.

**Fig 2:**
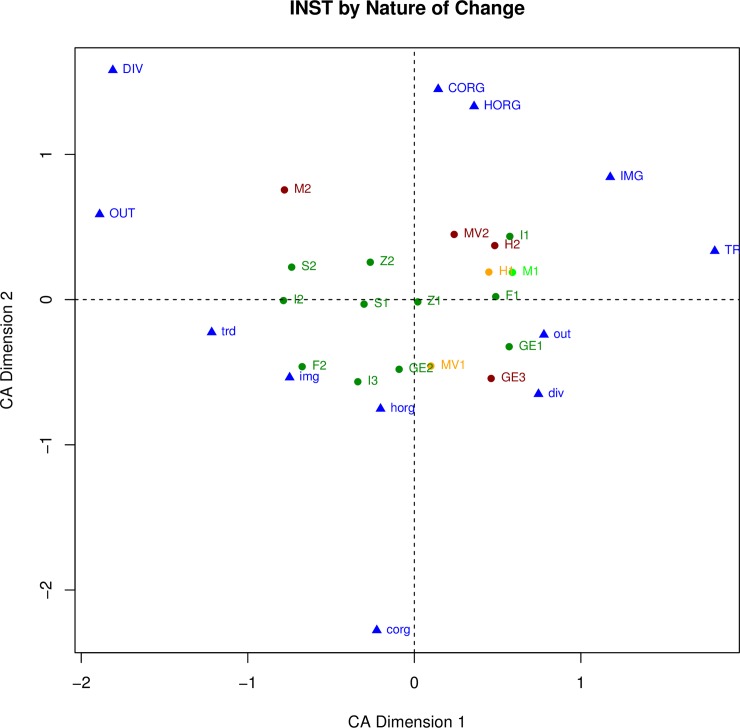
Correspondence analysis of all nature of change variables and the transformation cases. Transformation cases are color-coded by their loading on institutional breakdown.

**Fig 3:**
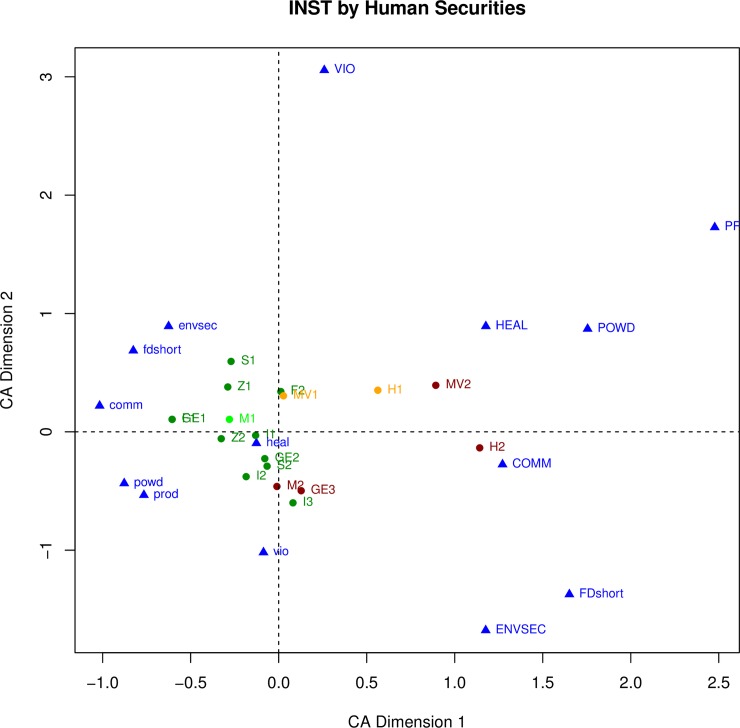
Correspondence analysis of all human securities variables and the transformation cases. Transformation cases are latter color-coded by their loading on institutional breakdown.

**Fig 4:**
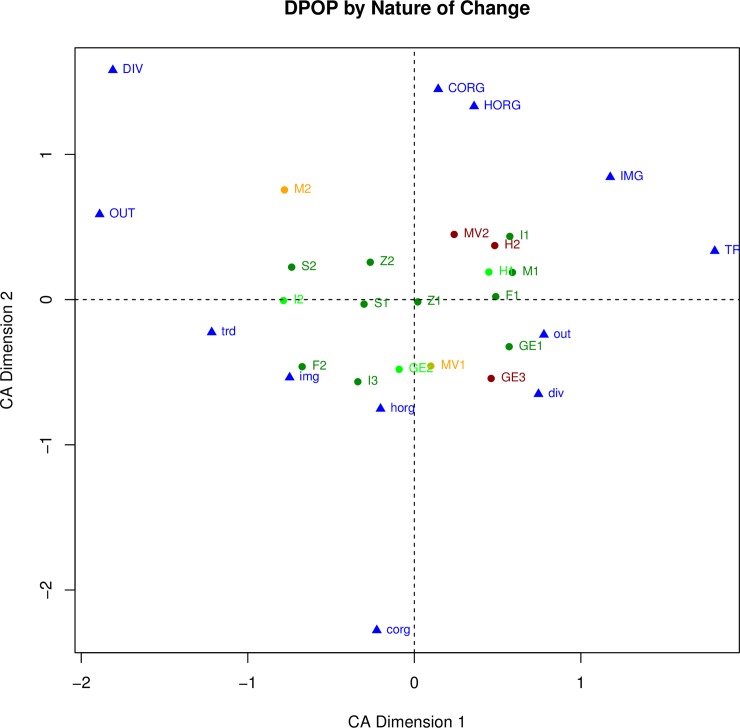
Correspondence analysis of all nature of change variables and the transformation cases. Transformation cases are color-coded by their loading on depopulation.

**Fig 5:**
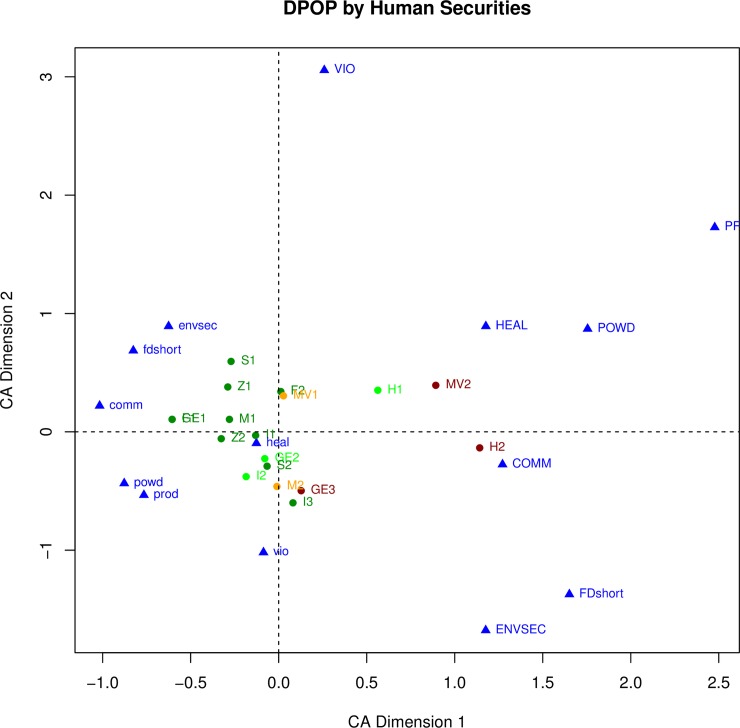
Correspondence analysis of all human securities variables and the transformation cases. Transformation cases are color-coded by their loading on depopulation.

We expect that depopulation and institutional breakdown will both be associated with other kinds of changes, including changes in human securities, but in different ways. For example, of the three transformation cases that ended cultural sequences with complete institutional breakdown and depopulation (INST = 1 and DPOP = 1) all three (**GE3, H2, MV2**) were associated with a decrease in food security (FDshort = 0.75 or 1). However only one (**MV2**) was associated with a severe decrease in personal security (i.e., an increase in violence) and was coded as VIO = 1; the other two (**GE3** and **H2**) did not exhibit evidence of violence and were coded as VIO = 0. To explore the relationships between the key variables and the other variables, each relationship is first examined separately, in Figs 2, 3, 4 and 5. For example, Fig 2 plots the six nature of change variables and the transformation cases, with the cases color-coded by their weighting on institutional breakdown. Red indicates an almost complete breakdown of institutions (INST = 1), gold indicates loss of many (INST = .75), bright green indicates loss of a few, and dark green indicating no institutional change (INST = 0). The same procedure is used to assess the relationships between the other pairings in the figures discussed in the paragraphs that follow (human securities variables and institutional breakdown in Fig 3, nature of change variables and depopulation in Fig 4, and human securities variables and depopulation in Fig 5).

The overall result shown by the Fig 2 plot is the highly variable relationship between the six nature of change variables and overall institutional change. We describe the results in some detail to illustrate the kinds of findings that can be discerned from these plots. The variables (indicated in blue) themselves are widely distributed, with positive loadings (capital letters) in both upper quadrants and negative loadings in both lower quadrants, indicating that they are quite different from one another, an observation reinforced by the complex patterning of the cases. Two variables, DIV (material culture diversity) and OUT (outside influence) plot fairly close to one another in the upper left, probably because they are affected by similar processes. Similarly, HORG (household organization) and CORG (community organization) plot near one another in the upper right, probably because they are often related. The eleven transformation cases that experienced no institutional change (INST = 0) are colored dark green and plot close together near the center of the graph, with some horizontal spread especially to the left. Both in-migration (IMG) and interregional trade (TRD) pull the cases towards the right, including the three Norse *Landnám* cases (**GE1, I1, F1**), for which IMG = 1. At the other end of the spread, **I2** and **S2** are both pulled toward the left having experienced a high degree of change in material culture diversity (DIV = 1 and 0.75) and increasing outside influence (OUT = 0.75). Of the four transformation cases that experienced institutional breakdown (INST = 1) colored red (**M2, MV2, H2, GE3)**, three are above the X-axis, generally pulled upward by the nature of change variables. Of these, **MV2** and **H2** plot close to one another, both having experienced major changes in community organization (CORG = 1) and interregional trade that would have made it more difficult to get outside goods (TRD = 0.75 and 1). In contrast, **M2** in the upper left quadrant was characterized by major changes in community organization (CORG = 1) but no changes in interregional trade (TRD = 0). **M2**, the end of the Mimbres Classic period, is the only red case left of the Y-axis, separated from the other cases by its high material culture diversity (DIV = 1) score. **M2** is also the only red case (those with INST = 1) that did not also have total depopulation (DPOP = 0.75 for **M2**. This complicated discussion indicates that the overall relationship between degree of institutional breakdown and the nature of change variables is complex and inconsistent.

The relationship between the human security variables and institutional breakdown is much more consistent, shown on Fig 3. The variables (in blue) plot near to one another, except for VIO (personal security). There is a general separation between the six cases that experienced institutional breakdown (INST = 0.75 or 1, colored orange or red) and those that saw little or no institutional change (INST = 0 or 0.25, colored dark or bright green). The former are pulled to the right by their positive loadings on the human securities variables (higher scores indicate a decline), the latter pulled to the left by their inverse variables. These results show the strong relationship between institutional breakdown and a decline in the human securities. The one surprising exception is personal security (VIO). Four cases (**Z1, S1, MV1, MV2**) had scores indicating a decline in personal security (VIO = 0.75 or 1), and while they all plot above the X axis, they do not otherwise stand out.

The same procedures are applied to the depopulation (DPOP) scores to understand how depopulation is associated with both the general nature of change and change in human securities. Fig 2, discussed above, plotted the nature of change variables color-coded by institutional breakdown. Fig 4 plots those same variables but this time color-coded by depopulation; placement of the variables and transformation cases is the same on both figures, they differ only in how they are color-coded. The main difference between the two plots is the distribution of cases with intermediate depopulation scores (DPOP = 0.25 or 0.75), colored bright green or orange. On Fig 2 there are only three of these, all right of the Y-axis; on Fig 4 there are five and they are found in all four quadrants.

Fig 5 plots the human securities variables color-coded by depopulation (this is the same Fig 3 but with different color-coding). This plot again shows a horizontal separation, with cases pulled left by their positive loadings on the human securities variables (indicating a decline in security) and pulled right by their negative loadings on those variables. All five orange and red cases (DPOP = 0.75 or 1) are on or right of the Y-axis and most of the green cases (DPOP = 0 or 0.25) are to the right. On this plot (in contrast to Fig 3) the first Hohokam transformation (**H1**) stands out as the only bright green case (DPOP = 0.25) pulled to the right. This case was characterized by little population change but negative changes in most human security variables as the regional system ended and population aggregated. This case is also unusual in that it had significant institutional breakdown in association with only minor depopulation (INST = 0.75, DPOP = 0.25), although its loading on INST does not affect its placement on this graph.

**Fig 6:**
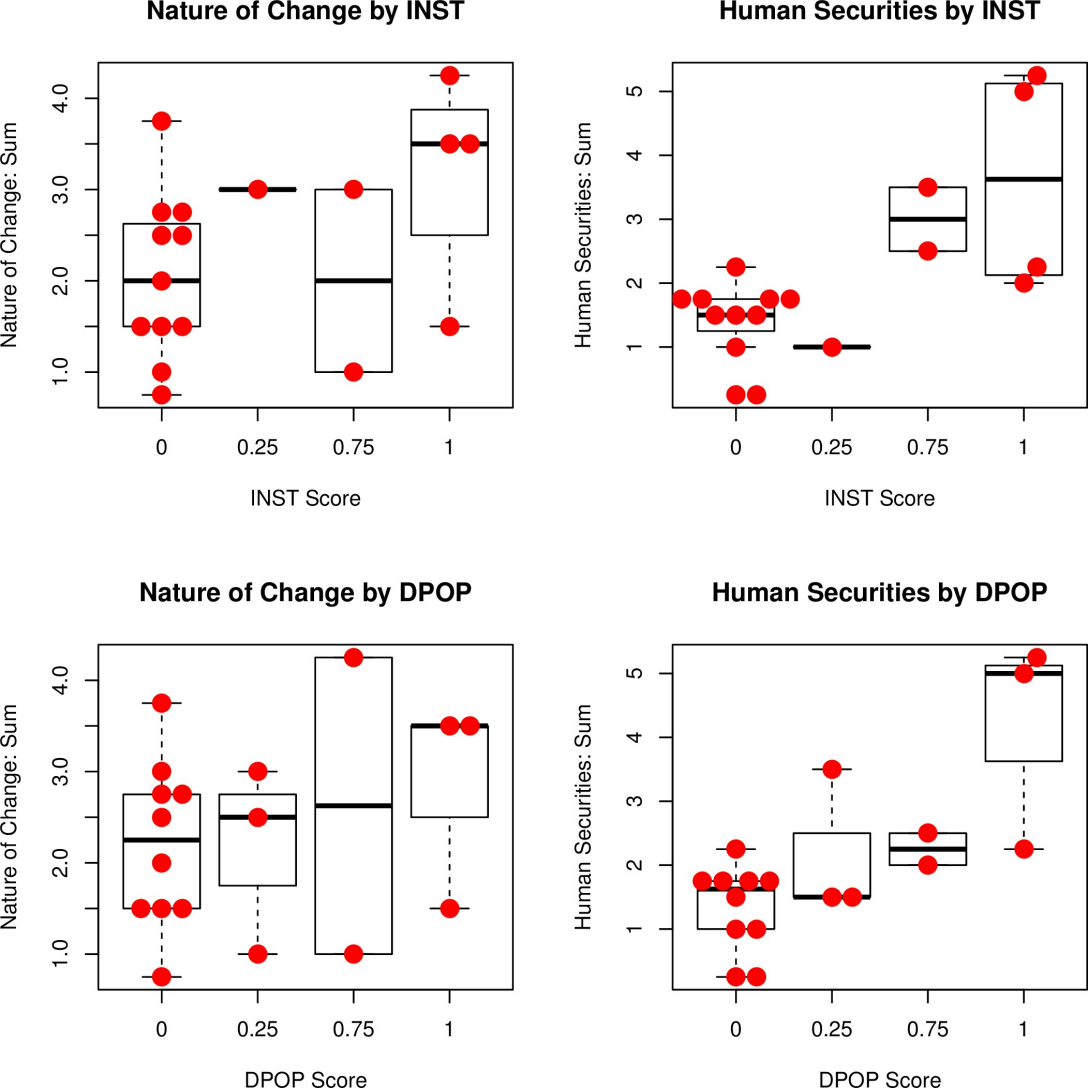
Box and dot plots summarizing the four analyses. These illustrate differences between institutional change and depopulation.

A comparison across these four analyses is facilitated by reproducing summary data in the box plots with overlaid dot plots, shown together on Fig 6. These show how the total scores on each set of variables (shown in Table 3) relate to institutional change or depopulation. For example, 6a in the upper left (drawing from the same data as Fig 2) shows the inconsistent relationship between the nature of change variables and institutional breakdown. Specifically, the eleven cases with no institutional breakdown (INST = 0) experienced varying degrees of change in other variables. The two extremes are from Iceland, with **I3** exhibiting only minor changes in material culture diversity, outside influence, and household organization (sum = 0.75) and **I1** exhibiting changes in all the variables except material culture diversity (sum 3.75). The four cases with institutional breakdown (INST = 1) also exhibit a wide spread though with a higher median and midrange.

Examination of the four plots shown in Fig 6 clarifies the pattern discerned in the correspondence analyses described above. There is an inconsistent relationship between transformations with high scores on the key variables (INST or DPOP = 0.75 or 1.) and the other nature of change variables, shown in 6a and 6c on the left side. This tells us that that change can take many forms. In contrast, transformations with high scores on the key variables are consistently associated with higher scores on the human securities variables (indicating a decline in security), shown in 6b and 6d on the right side. This says that some changes, especially collapses, are more difficult than others.

### Experiencing social transformations

The analyses presented so far considered the relationships among three sets of variables: the two key variables, institutional breakdown and depopulation; the six generally neutral nature of change variables; and the seven human security variables that track changes in the condition of life. While the nature of change variables do not pattern consistently, the human security variables are strongly associated with the key variables. Specifically, larger degrees of institutional and demographic change are associated with more declines in human security. In this final section we investigate this relationship in more detail, looking at the individual cases and their scores on each variable to better understand how and why some changes are more difficult than others (Goal #3).

Table 5 presents the data, with cases ordered by the sum of their scores on the two key variables, and the scores on the human security variables color-coded. The overall distribution reinforces the findings of the previous analysis, the association of scores on the key variables and human security variables. All four cases in which there was a high degree of institutional breakdown and depopulation (INST+DPOP = 1.75 or 2) also have high scores for the human security variables, indicating a decline in security. The cases with lower scores on the key variables are also major social transformations but of a different sort, involving, for example, a major change in settlement aggregation (**Z1** and **S1**) or administrative reorganization (**I2**). Importantly, these other kinds of changes involve much less decline in the human securities.

**Table 5:** Human security variable scores for all transformation cases.

			Human Security Variables							
Transformation	Key sum		FDshort	ENVSEC	PROD		COMM	VIO	HEAL	POWD
GE1	0		0	0	0		0	0.25	0	0
F1	0		0	0	0		0	0.25	0	0
Z2	0		0	0	0		0.75	0	0.25	0
I3	0		.75	1	0		0.25	0	0	0.25
F2	0		0	0	0.75		0	0	0	0.75
Z1	0		0	0	0		0.75	0.75	0.25	0
S1	0		0.25	0	0.25		0	1	0	0.25
S2	0		0.75	0.25	0		0.25	0	0.25	0.25
I1	0		0	0.75	0		0	0.25	0	0.75
GE2	0.25		0.75	0.25	0.25		0	0	0	0.25
MV1	1.5		0.25	0.25	0.25		0.75	0.75	0	0.25
I2	0.25		0	1	0		0.25	0	0	0.25
M1	0.25		0	0	0.25		0.25	0	0.25	0.25
H1	1		0.25	0.25	0.75		0.75	0.25	0.25	1
M2	1.75		0.25	0.75	0		1	0	0	0
GE3	2		1	0.25	0		1	0	0	0
H2	2		1	0.75	1		1	0	0.25	1
MV2	2		0.75	0.75	0.75		1	1	0.25	0.75

Cases in Table 5 are ordered and color-coded by their score on the two key variables (depopulation and institutional breakdown) summed.

Table 5 also reveals unexpected differences among the human security variables, in that some are strongly associated with INST and DPOP and others are not. The strongest associations are with food security (FDShort), environmental security (ENVSEC) and community security (COMM). In contrast, the other four variables (economic security, PROD; personal security, VIO, health security, HEAL, and power differences, POWD) pattern much less consistently. These associations are likely multi-causal. It is not surprising that the disintegration of communities (COMM = 1) is associated with the breakdown of institutions (INST = 1), although there are cases from other areas of the world where village and community life continues even as states and empires rise and fall [86,20]. The decline in community security noted in our cases makes the important point that major institutional changes often strongly affect the day-to-day lives of people throughout the society. In its discussion of community security, the UNDP notes that “Most people derive security from their membership in a group … that can provide a cultural identity and a reassuring set of values. Such groups also offer practical support” [46]. Loss of practical support would have negatively affected many aspects of people’s lives, including personal security and access to necessary food and economic resources [87,88]. The decline in food and environmental security at times of major change reinforces the difficulties caused by such changes, and of course the causality is not unidirectional—difficult conditions both contribute to and result from, major social transformations. For example, the end of the Mesa Verde sequence (**MV2**, coded as INST and DPOP = 1) is described as a difficult time in which competition, violence (VIO), and increasing inequality (POWD) exacerbated food insecurity (FDshort). Out-migration (DPOP) destabilized communities (COMM) and the difficult conditions were further exacerbated by drought conditions (ENVSEC). All of these factors, and the feedback among them, eventually led to tens of thousands of people leaving their homeland [89–93].

While the overall patterning is strong, the detailed results presented in Table 5 also point to cases in which difficult conditions were associated with little change in the key variables. In other words, while high scores for institutional breakdown and depopulation are consistently associated with a decline in the human securities, the converse is not true. In some of these cases, difficult conditions did not lead to more difficulties or breakdowns. For example, the third Iceland transformation (**I3)** was a difficult time in some ways, including severe winters and evidence of starvation [94], but these conditions and the society as a whole were managed with a new legal and administrative system [95]. In the second Salinas case **(S2)**, there was some decline in five of the human security variables but institutional stability. Overall, these results show that some societies are better able to withstand and manage difficult conditions [38,49], and understanding how that is accomplished could have important implications for today’s world.

## Summary, conclusions, and implications for today’s world

This research uses archaeological and some historical data to examine social transformations in the ancient past with focus on the human experience of living through those transformations. Social transformations are defined as lasting and major change in settlement, economy, and/or socio-political organization such that people’s life experiences before and after the transformation would different. Drawing on and synthesizing results from a large collaborative project, the research analyzes a total of 18 transformations from two areas of the world, the arid US Southwest and the cold and stormy North Atlantic. The transformations were analyzed based on expert knowledge, which allowed us to code changes in many variables. Data were analyzed using Qualitative Comparative Analysis (QCA) tools and techniques.

The research specifically looked at relationships among and within three sets of variables: (1) Two key variables, institutional breakdown and depopulation, which are emphasized in the literature on collapse. (2) Six nature of change variables, which describe changes in realms such as household organization or material culture diversity that are not necessarily desirable or undesirable. (3) Seven human securities variables, which describe changes in the conditions of life such as food security and violence.

The research as a whole, especially the analysis based on expert knowledge and the QCA techniques, accomplished our first goal. We developed and presented a method for systematically comparing different kinds of transformations and the human experience of those transformations in different kinds of settings based on different kinds of data.

The second goal was to consider the utility of a typology of social transformations. Can we define types or categories that encompass similar types? Overall, the answer is no. If the cases are sorted according to one variable, the resultant group has considerable variation in other variables. The one exception is the cases with high scores on the key variables that would generally be classified as “collapses.” Our general conclusion is that research would be best served by considering the range of variability in these wide-ranging social transformations.

The overarching purposes of this research were to explore the relationship between social transformations and people’s lived experiences (Goal #3) and consider the implications for today’s world (Goal #4). We address these goals by first summarizing five empirical conclusions relevant to Goal #3, and then conclude with the broader contemporary implications.

1. Some social transformations are more difficult than others. Our results, considering all cases and variables, are very clear: High scores on the key variables–institutional breakdown and depopulation–are associated with the greatest declines in the human securities. Social transformations, including collapses, *are* a normal part of the sweep of history, but many people who live through them experience difficult times. This conclusion is not surprising, but it reinforces the importance of looking at transformations at many scales, the human as well as the institutional and historical [96].
2. Conversely, some changes are less difficult than others. Our analysis began with the identification of major social transformations, which constitute our eighteen cases. Those cases that involve little or no institutional breakdown or depopulation were associated with few declines in human security. This points to the importance of the ability or willingness to make some changes now that might ameliorate a difficult situation and stave off collapse later. Whether it is called flexibility or resilience ([97] or avoidance of a rigidity trap [17], the need for these kinds of changes is widely recognized.
3. Community security deserves more emphasis. Of the seven human security variables we examined, the one most strongly associated with the key variables is community security. While this also is not surprising, it points to the importance of understanding the role of community in social change and especially as a means of providing support through difficult times. The variables food security and environmental security were also associated with the key variables; specifically, declines in food and environmental security often accompanied depopulation and institutional breakdown. It is possible that greater community security could have ameliorated these difficulties.
4. The relationships among the variables are complex and multi-causal. Certainly, institutional breakdown would have negatively impacted the human securities, but declining conditions of life would also have led to institutional breakdown and out-migration. Other research on some of these cases found that high vulnerability to food shortages is strongly associated with major social changes [25]. To a large extent, the archaeological literature has focused on explaining collapses and other social transformations, often as a result of difficult conditions including climate change, food insecurity and vulnerability to food shortage, warfare, changing interregional interactions, and increasing inequality (examples from the Southwest and North Atlantic include: [25,89,90,98–102]). We hope that the two perspectives will increasingly come together to understand what are likely recursive processes.
5. Some societies are better able to deal with difficulties than others. A recent analysis of violence in the Southwest compared two areas, the Central Mesa Verde region (from which our cases **MV1** and **MV2** are drawn) and the Northern Rio Grande [38], where many people settled after they left the Mesa Verde region. In the former, food insecurity is generally associated with violence but in the latter it is not. The authors concluded that the later societies–ancestral to Pueblo Peoples today–developed social and cultural norms, including enhanced community security, that kept violence in check and led to a more stable and prosperous society overall [49]. Our data support this hopeful scenario. There are cases in which declining human security did not lead to collapse but rather other kinds of social transformations that, in the longer term, may have ameliorated those declines. These kinds of cases, in the past as well as today’s world, are worthy of much more attention.

The Intergovernmental Panel on Climate Change (IPCC) just (October 2018) issued an alarming report [103]. Continued warming of the atmosphere will bring large-scale devastating consequences as early as 2040, inundating heavily populated coastlines, desertifying other areas, exacerbating poverty, and creating global-scale food shortages, among many other consequences. These enormous problems demand enormous responses if we are to have any hope of slowing climate change and averting the humanitarian and environmental disasters that will ensue if we do not. Some of these responses–the technologies of renewable energy, the economics of 21^st^ century global capitalism, politics in the age of social media–will be unique to our times. But other strategies are more universal, and archaeology, with its long-term perspective on how past societies faced major problems, can provide insights that are useful today, challenging current assumptions and offering examples of success and failure.

Our research found strong associations between community security–the mutual support among people in a community–and the key variables (point #3 above): In cases where community security was strong, there was less demographic decline and less institutional change. This is not an isolated finding. Our conclusions are consonant with those of archaeologists, cultural anthropologists, disaster managers, and others regarding the importance of local social factors in reducing people’s vulnerability to difficult conditions [25,58,104–107]. Specifically, using comparative archaeology, Peregrine concluded that people’s participation in governance increases society’s resilience to disasters [58]. Numerous studies demonstrate that social networks and strong communities contribute to people’s economic security (e.g., [87,88,108]). A recent review by an expert on vulnerability [109] found that social capital increases adaptive capacity and therefore resilience to the risks of climate change [110]. Another study drew on archaeological cases and sustainability science to show the importance of household-level food storage as means of reducing food insecurity even in a setting, contemporary India, with large-scale national-level food storage [111].

Furthermore, the association between strong community security and lesser degrees of demographic decline and institutional change is not a one-way process (point #4 above). To put it in negative terms, individuals’ vulnerability not only makes it more difficult for them to cope with difficult times, that vulnerability can also exacerbate and create difficulties in many arenas. The recent Global Report on Food Crisis 2018 documents a vicious cycle in which conflict drives food insecurity which in turn drives causes even more instability [112]. At a smaller scale, a number of archaeological cases document how apparently minor declines in conditions of life, such as the need to cultivate less than ideal fields, contribute to major institutional changes, including some characterized as “collapses” [102,113,114]. Conversely, decent conditions of life increase people’s capabilities, including the capability to participate in society in positive ways, possibly averting collapse [115–117].

This research, and the archaeology of the human experience approach in general [19], began with the premise that we *should* consider the local level and take people’s experiences into account because they matter: It is important to understand both large-scale social transformations and their effects on people’s lives. Our results, augmented by numerous other studies, point to a much stronger conclusion: We *must* consider the people’s experiences because what happens at the local level can stabilize society, can augment people’s capabilities for contributing in positive ways, and thus can help avert disaster. The implications for policy makers are clear. The enormous problems we face today absolutely demand enormous responses. However, enormous does not mean only “large scale.” Rather, an enormous response to climate change must be multi-scalar, encompassing everything from local-level networks and grass-roots activism to national and international programs.

## Supporting information

S1 Table Appendix. Justification for codes assigned for each variable for each case.
(DOCX)

S1 Text Coding examples. Examples of how the transformation cases were coded.
(DOCX)
