# Supplementary material for: The human experience of social transformation: Insights from comparative archaeology
Source: PLoS One. 2018 Nov 29;13(11):e0208060. doi: 10.1371/journal.pone.0208060 (PMC6264852; doi:10.1371/journal.pone.0208060)
Supplement: S1 Table — Justification for codes assigned for each variable for each case. (DOCX) [file pone.0208060.s001.docx]

**S1 Appendix.**

Justification for codes assigned for each variable for each case.

**Key Variables**

**INST - institutional breakdown**

***Was the transformation characterized by a breakdown of institutions resulting in a change in core traditions?*** *Loss of institutions is consonant with Tainter’s (1988) definition of “collapse.”****.***

**Yes** – Almost total loss of these institutions.

**More yes than no** – Loss of many but probably not all.

**More no than yes** – Loss of a few.

**No** – No negative change.

| **Transfor-mation** | **Dates (CE)** | ***INST*** | ***Explanation of code for INST*** |
| --- | --- | --- | --- |
| GE1 | 980-1000 | 0 | Landnám: Norse settlers/colonizers from Iceland transferred and continued existing institutions. Early churches, hierarchy presumably with large landholdings and dependent farms [1]. |
| GE2 | 1250-1310 | 0 | Recession: Introduction of Norwegian ecclesiastical and royal institutions [1]. |
| GE3 | 1400-1450/70 | 1 | End of Norse settlements: Decline and end of ecclesiastical and royal interest in Greenland [1,2]. |
| I1 | 870-890 | 0 | Norse Landnám: Norse settlers/colonizers transferred and continued existing institutions, shown in settlement data [3]. |
| I2 | 950-1000 | 0 | Consolidation: Continuity of institutions evidenced in assembly sites, early churches, and Norse and Christian symbolism [4]. |
| I3 | 1250-1300 | 0 | Economic and Political Threshold: New law code and new administrative system evident in historical documents [5]. |
| F1 | 800-850 | 0 | Norse Landnám: Settlers/colonizers transferred and continued existing institutions [6]. |
| F2 | 1250-1300 | 0 | Sociopolitical reorganization: New law code and new administrative system evident in historical documents [7]. |
| Z1 | 1250-1290 | 0 | Pueblo III – Pueblo IV: There is no evidence that institutions broke down in a way that reduced complexity and indeed this transition is associated with the creation of many new institutions [8]. |
| Z2 | 1350-1400 | 0 | Pueblo IV – Protohistoric: Although this transformation involved a move away from many large villages to new ones there is no evidence for a loss of institutions and perhaps even evidence for the creation of some new institutions in the wake of the arrival of new populations in the region [9,10]. |
| S1 | 1275-1325 | 0 | Jacal – Masonry: Development of new institutions (kivas in masonry pueblos [11–15]. |
| S2 | 1400-1425 | 0 | Late Pueblo: No change, possible increase in complexity [16,17]. |
| H1 | 1070-1100 | .75 | Sedentary – Classic: Collapse of regional system including ballcourts and markets [18,19]. |
| H2 | 1375-1450 | 1 | End of Classic: No continuity [20,21]. |
| M1 | 950-1000 | .25 | Pithouse – Classic: Mostly reinforcement of existing patterns but end of great kivas [22]. |
| M2 | 1130-1150+ | 1 | End of Classic: End of architectural and material culture traditions [23,24]. |
| MV1 | 880-920 | .75 | End of Pueblo I: New institutions (U-shaped roomblocks at Dolores) didn’t continue [25]; unit pueblo households replaced by pocket pithouses at Grass Mesa Village [26]. After hiatus, Pueblo I patterns generally continued/reproduced in Pueblo II. |
| MV2 | 1240-1290 | 1 | Pueblo III depopulation: End of occupation; probably deliberate rejection of existing institutions [27–29]. |

**DPOP - depopulation**

***Was the transformation characterized by regional scale depopulation or a high degree of population loss?***

**Yes** - Almost total population decline (ca. >75% of absolute regional population).

**More yes than no** - Regional population decline with some continuity (50% decline).

**More no than yes** - Population continuity with evidence for slight decline or stability.

**No** - Little to no evidence of changes in population or increasing regional population.

| **Transfor-mation** | **Dates (CE)** | ***DPOP*** | ***Explanation of code for DPOP*** |
| --- | --- | --- | --- |
| GE1 | 980-1000 | 0 | Landnám: N/A |
| GE2 | 1250-1310 | .75 | Recession: Marginal areas (for pastoralism) abandoned [30]. |
| GE3 | 1400-1450/70 | 1 | End of Norse settlements: Total depopulation. |
| I1 | 870-890 | 0 | Norse Landnám: Settlement data [3]. |
| I2 | 950-1000 | .25 | Consolidation: Internal settlement expansion and emigration to Greenland [31]. |
| I3 | 1250-1300 | 0 | Economic and Political Threshold: No evidence for depopulation [5]. |
| F1 | 800-850 | 0 | Norse Landnám: |
| F2 | 1250-1300 | 0 | Sociopolitical Reorganization: No evidence. |
| Z1 | 1250-1290 | 0 | Pueblo III – Pueblo IV: There is no evidence for depopulation and populations were likely increasing slightly across this transformation [8]. |
| Z2 | 1350-1400 | 0 | Pueblo IV – Protohistoric: There is little evidence for a depopulation across this transition [32]; this transition is marked by the arrival of migrants from outside of the region [10]. |
| S1 | 1275-1325 | 0 | Jacal – Masonry: Continuity [33]. |
| S2 | 1400-1425 | 0 | Late Pueblo: Continuity [33]. |
| H1 | 1070-1100 | .25 | Sedentary – Classic: Flat or slight decline in Phoenix Basin [34]. |
| H2 | 1375-1450 | 1 | End of Classic: Almost total depopulation [21,35]. |
| M1 | 950-1000 | 0 | Pithouse – Classic: Continuity and population growth [36]. |
| M2 | 1130-1150+ | .75 | End of Classic: Most people leave large villages, many leave region [36,37]. |
| MV1 | 880-920 | .75 | End of Pueblo I: Decline begins 880. Some areas (Dolores) mostly depopulated after 920, but occupation of other areas, esp. Mesa Verde, continues [26,38]. |
| MV2 | 1240-1290 | 1 | Pueblo III depopulation: 10s of thousands leave the region. No evidence of habitations after 1290 [28,38,39]. |

**Nature of Change Variables**

**IMG - in-migration**

***Was the transformation associated with evidence for the immigration of new individuals or groups into the region? This question is specifically referring to the arrival of people from distinct social traditions, not continued arrival of migrants from the homeland.***

**Yes** - Evidence for the establishment of new communities or substantial segments of existing communities by immigrants from outside the region.

**More yes than no** - Evidence for immigrants joining existing communities (or the establishment of new local/immigrant communities).

**More no than yes**  - Limited evidence for immigrants in the region with limited or uncertain impacts.

**No -** Little to no evidence for new immigrants in the region.

| **Transfor-mation** | **Dates (CE)** | ***IMG*** | ***Explanation of code for IMG*** |
| --- | --- | --- | --- |
| GE1 | 980-1000 | 1 | Landnám: Immigration from Iceland [40]. |
| GE2 | 1250-1310 | 0 | Recession: No signs of newcomers [41]. |
| GE3 | 1400-1450/70 | .25 | End of Norse settlements: Inuit arrive in Norse settlement very shortly after depopulation [42]. |
| I1 | 870-890 | 1 | Norse Landnám: Settlement data [3]. |
| I2 | 950-1000 | .25 | Consolidation: Isotopic evidence of immigrants [43]. |
| I3 | 1250-1300 | 0 | Economic and Political Threshold: No evidence [5]. |
| F1 | 800-850 | 1 | Norse Landnám: Settlement data [6]. |
| F2 | 1250-1300 | 0 | Sociopolitical Reorganization: No evidence. |
| Z1 | 1250-1290 | .25 | Pueblo III – Pueblo IV: There is little evidence for the arrival of migrants across most of the region but a few areas along the southern edge of Colorado have architectural and ceramic evidence suggesting the arrival of small numbers of non-locals [8,44]. |
| Z2 | 1350-1400 | .75 | Pueblo IV – Protohistoric: This transition was marked by the arrival of many non-local people (as evidence by artifact styles and biodistance data) who joined existing communities or newly established communities which also housed local populations [9,10,45]. |
| S1 | 1275-1325 | 0 | Jacal – Masonry: Continuity [33]. |
| S2 | 1400-1425 | 0 | Late Pueblo: Continuity [33]. |
| H1 | 1070-1100 | .25 | Sedentary – Classic: Consolidation in Phoenix probably involving some in-migration. |
| H2 | 1375-1450 | .25 | End of Classic: Some evidence for immigration even as communities are depopulated [21,46]. |
| M1 | 950-1000 | .75 | Pithouse – Classic: Big population increase probably due to in-migration indicated by rate of increase and new non-local technologies (e.g., pottery and hearth styles) [36,47]. |
| M2 | 1130-1150+ | .25 | End of Classic: No definitive evidence, but there was so much population movement and influx of new styles and material at this time it’s likely there was some in-migration. |
| MV1 | 880-920 | .25 | End of Pueblo I: Lots of mixing and diversity through Pueblo I period, probably continues in these last decades [48,49]. |
| MV2 | 1240-1290 | .75 | Pueblo III depopulation: Consolidation into central Mesa Verde in mid-late 13^th^ century including new people moving in from north [28]. |

**DIV – material culture diversity**

***Was the transformation associated with an increase in material cultural diversity either due to local production or potentially marking new or shifting networks of regional scale interaction and identity?***

**Yes -** Substantial increase in the diversity of material culture including both household items and rare objects obtained through exchange.

**More yes than no** – Increases in the diversity of material culture including primarily rare objects (i.e., exchange objects), with continuity in household material culture.

**More no than yes** - Slight increases in material diversity or the frequency of rare objects, but overall continuity.

**No** - No evidence for increasing diversity of material culture across the transformation.

| **Transfor-mation** | **Dates (CE)** | ***DIV*** | ***Explanation of code for DIV*** |
| --- | --- | --- | --- |
| GE1 | 980-1000 | 0 | Landnám: Settlers probably had access to less diverse range of materials than in homeland though perhaps adapted to local resources with new technologies. |
| GE2 | 1250-1310 | 0 | Recession: Decline in contacts with Europe would cause scarcity of vital import items, especially iron [1,50]. |
| GE3 | 1400-1450/70 | 0 | End of Norse Settlements: End of contacts with Europe would cause scarcity of vital imports [1]. |
| I1 | 870-890 | 0 | Norse Landnám: No evidence. |
| I2 | 950-1000 | 1 | Consolidation: Large amounts of imported materials [4]. |
| I3 | 1250-1300 | .25 | Economic and Political Threshold: Introduction of small amounts of pottery [51]. |
| F1 | 800-850 | 0 | Norse Landnám: Settlers probably had access to less diverse range of materials than in homeland though perhaps adapted to local resources with new technologies. |
| F2 | 1250-1300 | .25 | Sociopolitical Reorganization: Lacking archaeological evidence, but establishment of trade monopoly likely helped ensure access to key import goods [52]. |
| Z1 | 1250-1290 | 0 | Pueblo III – Pueblo IV: This transition was marked by a reduction in material diversity across multiple material classes including ceramics, domestic architectural features, and perishable materials [8]. |
| Z2 | 1350-1400 | .75 | Pueblo IV – Protohistoric: This transition was marked by an increase in material diversity in particular in ceramic wares and domestic architectural features [45,53]. |
| S1 | 1275-1325 | .25 | Jacal – Masonry: Slight increase in exchanged ceramics from the west but not a lot of these [11,54–56] |
| S2 | 1400-1425 | .75 | Late Pueblo: Importing Glaze A and some obsidian, indicating participation in the Rio Grande version of the Southwestern “Cult” [54–58]. |
| H1 | 1070-1100 | .25 | Sedentary – Classic: Changes in material culture and symbols; more rare objects but less access to material from collapsed regional system. |
| H2 | 1375-1450 | 0 | End of Classic: Change (Salado types) but overall decrease in diversity [59,60]. |
| M1 | 950-1000 | 0 | Pithouse – Classic: Decline in imports and use of Hohokam motifs [61]. Mimbres B/W pottery becomes more elaborate, but the overall style is homogeneous [62]. |
| M2 | 1130-1150+ | 1 | End of Classic: Big increase in diversity of architecture, features, and pottery [47,63]. |
| MV1 | 880-920 | 0 | End of Pueblo I: Had been experimentation but mostly pre-880; no evidence for this in last decades or through the transformation. |
| MV2 | 1240-1290 | .75 | Pueblo III depopulation: Lots of experimentation esp. with public architecture [28]; increasing conformity but also elaboration in ceramic designs and diversity in vessel form. |

**OUT *–* outside influence**

***Was the transformation associated with increasing outside influence in the study area?***

**Yes -** Transformation was marked by dramatic increase in contact with and increased political integration into outside areas

**More yes than no** - Transformation associated with some increase contact with outside areas but little evidence of strong political relationships.

**More no than yes** - Transformation associated with small changes in the degree of outside influence but largely continuity.

**No -** No evidence for changes in the nature of outside influence across the transformation

| **Transfor-mation** | **Dates (CE)** | ***OUT*** | ***Explanation of code for OUT*** |
| --- | --- | --- | --- |
| GE1 | 980-1000 | 0 | Landnám: N/A |
| GE2 | 1250-1310 | 0,25 | Recession: Largely administered locally, but increased Norwegian royal and ecclesiastical influence [1]. |
| GE3 | 1400-1450/70 | 0 | End of Norse Settlements: End of Norwegian influence. |
| I1 | 870-890 | .25 | Norse Landnám: Lots of back and forth. |
| I2 | 950-1000 | .75 | Consolidation: Adoption of external symbols [4]. |
| I3 | 1250-1300 | .25 | Economic and Political Threshold: Royal control but with continuity of personnel [5]. |
| F1 | 800-850 | .25 | Norse Landnám: Lots of back and forth. |
| F2 | 1250-1300 | 1 | Sociopolitical Reorganization: Royal control, though largely administered locally [7,52]. |
| Z1 | 1250-1290 | 0 | Pueblo III – Pueblo IV: The transition was marked by a reduction in evidence for connections to outside areas (considering ceramics in particular) [8,64]. |
| Z2 | 1350-1400 | .25 | Pueblo IV – Protohistoric: This transition was marked by a slight increase in evidence associated with contact with outside areas including small numbers of Hopi Yellow Ware ceramics and an increasing frequency of obsidian [10,45]. |
| S1 | 1275-1325 | .25 | Jacal – Masonry: Evidence of external exchange is limited; marked increase in conflict but source is unclear; adoption of kivas signifies a shift in the nature of community and a shift away from engagement with Jornada Mogollon and towards Rio Grande [13,54–56,65]. |
| S2 | 1400-1425 | .75 | Late Pueblo: fully participating in the SW cult, exchange ties in Rio Grande area increasing Grande [54–56]. |
| H1 | 1070-1100 | 0 | Sedentary – Classic: Collapse of regional system indicates less outside influence. |
| H2 | 1375-1450 | .25 | End of Classic: Some in-migration [46]. |
| M1 | 950-1000 | 0 | Pithouse – Classic: If anything, less outside influence and increased isolation (decline in Hohokam motifs on pottery [61]. |
| M2 | 1130-1150+ | 1 | End of Classic: Large increases in inter-regional interaction, what was homogeneous region becomes part of several different traditions [66]. |
| MV1 | 880-920 | 0 | End of Pueblo I: Mostly people leaving, no evidence of outside influence. |
| MV2 | 1240-1290 | 0 | Pueblo III depopulation: Increasingly insular, though some back&forth through migration stream [29]. |

**HORG – household organization**

***Was the transformation associated with substantial changes in household scale social organization [using architecture as a proxy] (e.g., changes in household composition or size)?***

**Yes -** Evidence for major changes in household organization potentially including substantial changes in household composition, organization, or architectural forms associated with households.

**More yes than no** - Evidence for changes in household organization with some continuity. For example, changes in household size (scale) but not form.

**More no than yes** - Evidence for limited changes in household organization potentially including slight changes in room layouts without major changes in social organization.

**No** - Little to no evidence for change in household organization.

| **Transfor-mation** | **Dates (CE)** | ***HORG*** | ***Explanation of code for HORG*** |
| --- | --- | --- | --- |
| GE1 | 980-1000 | 0 | Landnám: As in homeland (Iceland). |
| GE2 | 1250-1310 | .25 | Recession: Centralized farms with dwelling and economy buildings in one large complex (middle to small sized farms) No changes of the layout of elite farms [67]. |
| GE3 | 1400-1450/70 | .25 | End of Norse settlements: Dwellings and economic buildings reduced in size. |
| I1 | 870-890 | .75 | Norse Landnám: Different architecture from homeland [68]. |
| I2 | 950-1000 | .25 | Consolidation: Change in layouts of farmhouses [69]. |
| I3 | 1250-1300 | .25 | Economic and Political Threshold: Another change in the layout of farmhouses [70]. |
| F1 | 800-850 | 0 | Norse Landnám: Earliest structures & settlement layout are same as in Norway [71]. |
| F2 | 1250-1300 | .25 | Sociopolitical Reorganization: Changes are related more to landscape organization & use (e.g. abandonment of shieling sites) than at the household scale [7]. |
| Z1 | 1250-1290 | .25 | Pueblo III – Pueblo IV: This transition was associated with slight changes in the organization of households associated with the movement from small structures to large ladder-constructed pueblos with less differentiation among rooms [8]. |
| Z2 | 1350-1400 | .25 | Pueblo IV – Protohistoric: This transition was marked by small changes in the organization of domestic space including a change in hearth placement and a greater diversity of room sizes when compared to the PIV period [8]. |
| S1 | 1275-1325 | 0 | Jacal – Masonry: The same number of rooms between jacal and masonry villages [11,13] suggests that households probably remain organized as they had been before. |
| S2 | 1400-1425 | 0 | Late Pueblo: Room sizes in early and late masonry sites are exactly the same, as is hearth orientation (data collected for LTVTP project; <https://core.tdar.org/collection/14044/long-term-vulnerability-and-transformation-project-ltvtp-documents-and-data>). |
| H1 | 1070-1100 | .75 | Sedentary – Classic: Major shift in household architecture, some continuity in overall organization [72,73]. |
| H2 | 1375-1450 | 1 | End of Classic: No continuity. |
| M1 | 950-1000 | .75 | Pithouse – Classic: Change and increased diversity in household architecture, new domestic architecture form; some now have separate storage rooms [63,74]. Inter-site variation in growth suggests differences in social organization [75]. |
| M2 | 1130-1150+ | 1 | End of Classic: New kind of more homogenous (single room) household architecture, probably more mobility [63]. |
| MV1 | 880-920 | .25 | End of Pueblo I: Non-continuity of U-shaped roomblocks, but these were rare. General continuity of early unit pueblo architecture. |
| MV2 | 1240-1290 | 1 | Pueblo III depopulation: Shifts in village layout that changes the organization of households in the late 13^th^ century [28,76], and new forms of architecture after the migration [29]. |

**CORG – community organization**

***Was the transformation associated with substantial changes in community scale social organization [using architecture as a proxy] (e.g., changes in community composition, organization, or size)? Note that this question refers to the organization of communities and not population movements involving the establishment of new settlements***

**Yes -** Evidence for major changes in community organization potentially including the creation of new forms/scales of community architecture, new layouts, etc.

**More yes than no** - Evidence for changes in community organization with some continuity. For example, changes in village size (scale) but not form.

**More no than yes** - Evidence for limited changes in community organization potentially including slight changes in layouts or size without apparent major changes in social organization

**No** - Little to no evidence for change in community organization.

| **Transfor-mation** | **Dates (CE)** | ***CORG*** | ***Explanation of code for CORG*** |
| --- | --- | --- | --- |
| GE1 | 980-1000 | .25 | Landnám: Overall continuity from homeland with some changes, esp. in size. |
| GE2 | 1250-1310 | .25 | Recession: Centralization of authority [1]. |
| GE3 | 1400-1450/70 | 0 | End of Norse settlements: No change that we can see. |
| I1 | 870-890 | 1 | Norse Landnám: Establish new communities, possibly new kinds of communities. |
| I2 | 950-1000 | .25 | Consolidation: Change in farm layouts and some relocations, begin building churches [69,77]. |
| I3 | 1250-1300 | 0 | Economic and Political Threshold: No evidence of change [5]. |
| F1 | 800-850 | .75 | Norse Landnám: Establishment of new communities. |
| F2 | 1250-1300 | 0 | Sociopolitical Reorganization: No evidence of structural or community organization change, but there was legislative and economic change [52,78]. |
| Z1 | 1250-1290 | 1 | Pueblo III – Pueblo IV: This transformation was associated with the creation of a new form of planned and rapidly constructed nucleated settlement that dramatically changed the organization of communities and substantially increased their scale [8]. |
| Z2 | 1350-1400 | .75 | Pueblo IV – Protohistoric: This transformation saw the creation of new communities similar in scale to before the transition but which were accretionally constructed and lacked evidence for a high level of community planning [8,9]. |
| S1 | 1275-1325 | 1 | Jacal – Masonry: Community configuration changed dramatically from dispersed hamlets and roomblocks to fully enclosed pueblos containing one or more plazas and one or more kivas [11,13–15]. |
| S2 | 1400-1425 | 1 | Late Pueblo: Communities were several times larger than previously suggesting that significant changes in community organization must have taken place, but we have little direct data on this. |
| H1 | 1070-1100 | .75 | Sedentary – Classic: Change from pithouse to compound architecture but some continuity in organization. Shift from ballcourts (at most large sites) to platform mounds (more exclusive) [79–81]. |
| H2 | 1375-1450 | 1 | End of Classic: Communities dissolve. |
| M1 | 950-1000 | .75 | Pithouse – Classic: Houses are now attached in room blocks rather than freestanding pithouses. Many villages are in the same places with *roughly* the same spatial organization but with informal plaza-like areas and without great kivas[82]. |
| M2 | 1130-1150+ | 1 | End of Classic: Most villages depopulated. Some people shift to hamlets which lack plazas or any ritual architecture [83]. |
| MV1 | 880-920 | .25 | End of Pueblo I: Dissolution of earlier large communities but general structure (roomblocks consisting of unit pueblos) continues. |
| MV2 | 1240-1290 | 1 | Pueblo III depopulation: Communities continue but with major reorganization and shifting locations both in the late 13^th^ century and after the migration. |

***TRD* – interregional trade**

***Was there a change in interregional trade that would have made it more difficult to get important goods?***

**Yes -** Important networks disappear.

**More yes than no -** Important networks changed making it more difficult to access goods.

**More no than yes -** Important networks changed in a way that might have been disruptive to access.

**No -** No change in interregional trade.

| **Transfor-mation** | **Dates (CE)** | ***TRD*** | ***Explanation of code for TRD*** |
| --- | --- | --- | --- |
| GE1 | 980-1000 | .75 | Landnám: More difficult to get goods in new place. |
| GE2 | 1250-1310 | .25 | Recession: Norwegian trade monopoly. Declining contacts – political changes in Scandinavia, climate changes [1,50]. |
| GE3 | 1400-1450/70 | 1 | End of Norse settlements: Connections to Europe lost [1,50]. |
| I1 | 870-890 | .75 | Norse Landnám: More difficult to get goods in new place. |
| I2 | 950-1000 | 0 | Consolidation: Increase in contact [5,68]. |
| I3 | 1250-1300 | 0 | Economic and Political Threshold: Increase in contact [5,68]. |
| F1 | 800-850 | .75 | Norse Landnám: More difficult to get goods in new place. |
| F2 | 1250-1300 | 0 | Sociopolitical Reorganization: Norway trade monopoly possibly increased Faroese access to key imports [52]. |
| Z1 | 1250-1290 | 0 | Pueblo III – Pueblo IV: There is very little direct evidence for substantial amounts of inter-regional trade before or after this transformation [8]. |
| Z2 | 1350-1400 | 0 | Pueblo IV – Protohistoric: There is overall little evidence for external trade before or after this transformation but it was likely increasing slightly across this transition with the arrival of new sources of obsidian and Hopi region ceramics [8,84]. |
| S1 | 1275-1325 | 0 | Jacal – Masonry: Trade actually increased slightly (see above) |
| S2 | 1400-1425 | 0 | Late Pueblo: Trade increased [16,55,56,58]. |
| H1 | 1070-1100 | 1 | Sedentary – Classic: End of regional system [20,59,60]. |
| H2 | 1375-1450 | 1 | End of Classic: Increasingly shut off. |
| M1 | 950-1000 | .75 | Pithouse – Classic: Classic is increasingly isolated, cut off from earlier interaction with Hohokam [61]. |
| M2 | 1130-1150+ | 0 | End of Classic: Inter-regional interaction increases substantially. |
| MV1 | 880-920 | .25 | End of Pueblo I: Decline in red ware (moved from western to central part of the region, not really inter-regional). |
| MV2 | 1240-1290 | .75 | Pueblo III depopulation: Increasing isolation. |

**Human Securities Variables**

**FDShort – Food Security**

***Is there evidence of a decline in the availability of food, for at least some sector of society?***

**Yes -** Increase in nutritional deficiencies for at least some sector of society.

**More yes than no -** Decline in important resources but little or no evidence of increased malnutrition.

**More no than yes** - Might have had to work harder and/or use alternative resources, but no evidence of actual food shortages or malnutrition.

**No –** No change in food availability.

| **Transfor-mation** | **Dates (CE)** | ***Fdshort*** | ***Explanation of code for FDShort*** |
| --- | --- | --- | --- |
| GE1 | 980-1000 | 0 | Landnám: Subsistence based on pastoralism and seal (Arneborg et al. 2012). |
| GE2 | 1250-1310 | .75 | Recession: Decline in terrestrial resources, increased dependence on seal – no nutritional deficiencies recorded [41,85]. |
| GE3 | 1400-1450/70 | 1 | End of Norse settlements: Decline in terrestrial resources, increased dependence on seal – no nutritional deficiencies recorded [41,85]. |
| I1 | 870-890 | 0 | Norse Landnám: No evidence of decline in availability of food [86]. |
| I2 | 950-1000 | 0 | Consolidation: No evidence of decline in availability of food [87]. |
| I3 | 1250-1300 | .75 | Economic and Political Threshold: Severe winters, evidence of starvation [88]. |
| F1 | 800-850 | 0 | Norse Landnám: No evidence of decline. |
| F2 | 1250-1300 | 0 | Sociopolitical Reorganization: No evidence of decline. |
| Z1 | 1250-1290 | 0 | Pueblo III – Pueblo IV: Food resources used and their availability appear to change little across the transformation (ethnobotanical data; <https://core.tdar.org/dataset/405543/carp-macrobotanical-database>). . |
| Z2 | 1350-1400 | 0 | Pueblo IV – Protohistoric: Food resources and their availability appear to change little across the transformation (ethnobotanical data; <https://core.tdar.org/dataset/405543/carp-macrobotanical-database>). |
| S1 | 1275-1325 | .25 | Jacal – Masonry: There is no direct evidence for a decline in the food supply. Storage rooms full of corn are burned during this time, however [65,89], and the landscape appears to have been hostile to some degree possibly making hunting, as well as farming, a challenge. |
| S2 | 1400-1425 | .75 | Late Pueblo: Faunal data document a marked reduction in the local availability of antelope (resource depression), suggesting a decline in access to meat [90–92]. |
| H1 | 1070-1100 | .25 | Sedentary – Classic: End of regional system may have cut off their access to some resources, including deer [93,94]. |
| H2 | 1375-1450 | 1 | End of Classic: Different studies reach different conclusions about biological evidence of real nutritional stress [95–97]; collapse of irrigation system would have decreased availability of food. |
| M1 | 950-1000 | 0 | Pithouse – Classic: Good time, no evidence of problems. |
| M2 | 1130-1150+ | .25 | End of Classic: May have had to work harder [98] but no evidence of actual shortage. |
| MV1 | 880-920 | .25 | End of Pueblo I: Possible stress on resources but no real evidence of shortages. |
| MV2 | 1240-1290 | .75 | Pueblo III depopulation: Decline in deer possibly replaced by turkey [99]; restricted access to productive land because of violence. |

**ENVSEC – environmental security**

***Was there a decline in environmental security?***

**Yes** - Irreversible or long term degradation (beyond only resource depletion).

**More yes than no** - Some evidence of degradation, but mostly resource depletion.

**More no than yes** - Resource depletion but no evidence of long-term environmental degradation.

**No** - No evidence of either resource or overall environmental degradation.

| **Transfor-mation** | **Dates (CE)** | ***ENVSEC*** | ***Explanation of code for ENVSEC*** |
| --- | --- | --- | --- |
| GE1 | 980-1000 | 0 | Landnám: Virgin soils. Evidence of soil erosion and vegetation change based on lake sediments to about c. 1230 [100]. |
| GE2 | 1250-1310 | .25 | Recession: From c. 1230 decrease in the rate of erosion probably due to decline in agro-pastoral activities [100]. |
| GE3 | 1400-1450/70 | .25 | End of Norse settlements: Pressure easing on some resources. End of harbor seals because of sea ice [88]. |
| I1 | 870-890 | .75 | Norse Landnám: Immediate impact (kill off walrus); soil erosion and deforestation begin [101]. |
| I2 | 950-1000 | 1 | Consolidation: Soil erosion continues but it is not economically significant [101]. |
| I3 | 1250-1300 | 1 | Economic and Political Threshold: Soil erosion continues but it is not economically significant [101]. |
| F1 | 800-850 | 0 | Norse Landnám: Limited impact of landscape [102]. |
| F2 | 1250-1300 | 0 | Sociopolitical Reorganization: No resource depletion; well established management of pastures [103]. |
| Z1 | 1250-1290 | 0 | Pueblo III – Pueblo IV: No evidence for resource depletion across this transformation [104]. |
| Z2 | 1350-1400 | 0 | Pueblo IV – Protohistoric: No evidence for resource depletion across this transformation [104]. |
| S1 | 1275-1325 | 0 | Jacal – Masonry: No direct data on environmental change, but no indication that environmental security declined |
| S2 | 1400-1425 | .25 | Late Pueblo: Resource depression in artiodactyls. Marked investment in modifying the landscape to improve crop production [105]. |
| H1 | 1070-1100 | .25 | Sedentary – Classic: Artiodactyls depleted [93,94]; possible downcutting of Gila R. [106]. |
| H2 | 1375-1450 | .75 | End of Classic: Loss of upland resources [93,107]; soils degraded in irrigated areas along Salt R. [108]. |
| M1 | 950-1000 | 0 | Pithouse – Classic: Good times. |
| M2 | 1130-1150+ | .75 | End of Classic: Depletion of riparian areas [109] and soil degradation [110] in Mimbres Valley; in other parts of region little depletion [111] except artiodactyls [98]. |
| MV1 | 880-920 | .25 | End of Pueblo I: Reduction of woodlands and change in fuel use [112]. |
| MV2 | 1240-1290 | .75 | Pueblo III depopulation: Depletion of deer (possibly replaced by turkey [99]; changes to landscape indicated by fuel resources [113]. But environment may have recovered some from Pueblo II decline [38]. |

**PROD – economic security, production**

***Is there a change that increasingly alienated people from their means of production (land, boats, tools, irrigation networks, etc.)?***

**Yes** - Clear evidence of alienation from means of production across transformation (decreased access to land, moats, tools, etc).

**More yes than no -** Some evidence for alienation and people still maintain some access to their means of production.

**More no than yes -** Little evidence for alienation and most people maintain access to their means of production.

**No -** No evidence.

| **Transfor-mation** | **Dates (CE)** | ***PROD*** | ***Explanation of code for PROD*** |
| --- | --- | --- | --- |
| GE1 | 980-1000 | 0 | Landnám: Access to new resources. |
| GE2 | 1250-1310 | .25 | Recession: Boats and for the walrus hunt in the High Arctic on the hands of the elite farmers – trade central function connected to elite farmers [1,114]. |
| GE3 | 1400-1450/70 | 0 | End of Norse settlements: No evidence. |
| I1 | 870-890 | 0 | Norse Landnám: Some classes are alienated, but this doesn’t change [5]. |
| I2 | 950-1000 | 0 | Consolidation: Some classes are alienated, but this doesn’t change (Karlsson 2000). |
| I3 | 1250-1300 | 0 | Economic and Political Threshold: Some classes are alienated, but this doesn’t change [5]. |
| F1 | 800-850 | 0 | Norse Landnám: No evidence of change. |
| F2 | 1250-1300 | .75 | Sociopolitical Reorganization: There were always restrictions but these may have increased especially re. ownership to land [7]. |
| Z1 | 1250-1290 | 0 | Pueblo III – Pueblo IV: No evidence people were alienated from means of production. |
| Z2 | 1350-1400 | 0 | Pueblo IV – Protohistoric: No evidence people were alienated from means of production. |
| S1 | 1275-1325 | .25 | Jacal – Masonry: Fairly intense attacks on masonry villages suggest that it may have been risky to venture out on the landscape for farming, hunting and gathering, but we have no direct data regarding changes in resource access between the jacal and early masonry periods [65,89]. |
| S2 | 1400-1425 | 0 | Late Pueblo: There are no indications that resource access was restricted. |
| H1 | 1070-1100 | .75 | Sedentary – Classic: Platform mounds at headgates indicates increasing control over irrigation water and arable land [115]. |
| H2 | 1375-1450 | 1 | End of Classic: People lose access to their irrigation and land. |
| M1 | 950-1000 | .25 | Pithouse – Classic: First-comers’ claim to land [23] may have caused some restrictions on access |
| M2 | 1130-1150+ | 0 | End of Classic: Access probably became more open with regional reorganization. |
| MV1 | 880-920 | .25 | End of Pueblo I: Violence may have limited access to land but little evidence. |
| MV2 | 1240-1290 | .75 | Pueblo III depopulation: Filling in of landscape and violence would restrict access to good arable land (possibly analogous to recent famines in Africa). |

**COMM – community security**

***Did communities disintegrate or disappear?***

**Yes -** Communities were present and they do not continue beyond the transformation.

**More yes than no -** Communities reorganize, but maintain some of their former ties.

**More no than yes -** There are changes but the overall groupings (clusters) stay the same.

**No -** No evidence for change in communities.

| **Transfor-mation** | **Dates (CE)** | ***COMM*** | ***Explanation of code for COMM*** |
| --- | --- | --- | --- |
| GE1 | 980-1000 | 0 | Landnám: New communities established. |
| GE2 | 1250-1310 | 0 | Recession: Stability |
| GE3 | 1400-1450/70 | 1 | Depopulation: Communities end |
| I1 | 870-890 | 0 | Norse Landnám: New communities established [3]. |
| I2 | 950-1000 | .25 | Consolidation: Minor reduction in number of farms. |
| I3 | 1250-1300 | .25 | Economic and Political Threshold: Minor reduction in number of farms. |
| F1 | 800-850 | 0 | Norse Landnám: New communities established. |
| F2 | 1250-1300 | 0 | Sociopolitical Reorganization: Changes to landscape management [7], but no evidence of community change. |
| Z1 | 1250-1290 | .75 | Pueblo III – Pueblo IV: Communities change in form and size dramatically across this transition but ceramic exchange and measures of material similarity suggest that connections among communities were largely maintained [8]. |
| Z2 | 1350-1400 | .75 | Pueblo IV – Protohistoric: Communities change in form across this transition but connections within and between regions are largely maintained[9,45]. |
| S1 | 1275-1325 | 0 | Jacal – Masonry: Strong demographic continuity between jacal and early masonry villages indicates persistence of communities [11,13]. |
| S2 | 1400-1425 | .25 | Late Pueblo: Strong demographic continuity between early and late masonry pueblos [33] suggests that the same people were aggregating into larger villages. |
| H1 | 1070-1100 | .75 | Sedentary – Classic: Some communities (Snaketown) end, others (e.g., Pueblo Grande) gain population. |
| H2 | 1375-1450 | 1 | End of Classic: Communities leave the Phoenix Basin. Depopulation. |
| M1 | 950-1000 | .25 | Pithouse – Classic: Communities continue, but with some reorganization [116]. |
| M2 | 1130-1150+ | 1 | End of Classic: Village communities mostly depopulated [24,83]. |
| MV1 | 880-920 | .75 | End of Pueblo I: Large villages dissolve after 880 but there is some continuity in some places (on Mesa Verde). |
| MV2 | 1240-1290 | 1 | Pueblo III depopulation: Communities continue, possibly becoming more independent, until the final depopulation, but then dissolve. |

**VIO – personal security**

***Was there an increase in violence?***

**Yes -** Increased violence with definite evidence (skeletal trauma) of people being directly affected.

**More yes than no -** Indications that violence increased but evidence is less direct (e.g., burning).

**More no than yes -** Some indication of increase in competition (e.g., no man’s lands), but not direct evidence of personal violence.

**No -** No evidence of an increase in violence.

| **Transfor-mation** | **Dates (CE)** | ***VIO*** | ***Explanation of code for VIO*** |
| --- | --- | --- | --- |
| GE1 | 980-1000 | .25 | Landnám: Little evidence for violence on skeletons, frontier situation [41]. |
| GE2 | 1250-1310 | 0 | Recession: No evidence [41]. |
| GE3 | 1400-1450/70 | 0 | End of Norse settlements: Same [41]. |
| I1 | 870-890 | .25 | Norse Landnám: Frontier situation [5]. |
| I2 | 950-1000 | 0 | Consolidation: Continuing violence but no change [5]. |
| I3 | 1250-1300 | 0 | Economic and Political Threshold: Continuing violence but no change [5]. |
| F1 | 800-850 | .25 | Norse Landnám: Frontier situation. |
| F2 | 1250-1300 | 0 | Sociopolitical Reorganization: No evidence. |
| Z1 | 1250-1290 | .75 | Pueblo III – Pueblo IV: Evidence of violence in the form of room burning, defensive location of some sites, and skeletal evidence of violence at a small number of sites along the edges of the region [8,44]. |
| Z2 | 1350-1400 | 0 | Pueblo IV – Protohistoric: No direct evidence of violence or changes in the levels of violence across this transition. |
| S1 | 1275-1325 | 1 | Jacal – Masonry: Pueblos are burned, including the corn in storage; at least one is refortified [65,89]. |
| S2 | 1400-1425 | 0 | Late Pueblo: There is no evidence for violence in the late prehistoric pueblo mortuary or architectural records. |
| H1 | 1070-1100 | .25 | Sedentary – Classic: Emptying of landscape, creation of no-mans lands [117]. |
| H2 | 1375-1450 | 0 | End of Classic: Competition during Classic probably winding down by the end. |
| M1 | 950-1000 | 0 | Pithouse – Classic: No evidence of violence. |
| M2 | 1130-1150+ | 0 | End of Classic: No evidence of violence. |
| MV1 | 880-920 | .75 | End of Pueblo I: There had been some terrible violence earlier in Pueblo I [118]. Breakdown of McPhee Village ca. 880 includes burning and possibly interpersonal violence [25,49]. Stockades at some sites. |
| MV2 | 1240-1290 | 1 | Pueblo III depopulation: Violence escalated across 13^th^ century [119]; massacres at some late sites [99]. |

**HEAL – health security**

***Was there a decrease in health from causes other than nutritional deficiencies (epidemics, the plague, etc)?***

**Yes -** Definite indications of major increase in disease (e.g., epidemic).

**More yes than no -**  Reason to think there is an increase in disease, but evidence is less clear.

**More no than yes -** The possibility for the spread of diseases is clear (e.g., big aggregation) but no real evidence.

**No -** No evidence for a decrease in health.

| **Transfor-mation** | **Dates (CE)** | ***HEAL*** | ***Explanation of code for HEAL*** |
| --- | --- | --- | --- |
| GE1 | 980-1000 | 0 | Landnám: Stable [41]. |
| GE2 | 1250-1310 | 0 | Recession: Stable [41]. |
| GE3 | 1400-1450/70 | 0 | End of Norse settlements: Stable [41]. |
| I1 | 870-890 | 0 | Norse Landnám: No evidence. |
| I2 | 950-1000 | 0 | Consolidation: No evidence. |
| I3 | 1250-1300 | 0 | Economic and Political Threshold: No evidence. |
| F1 | 800-850 | 0 | Norse Landnám: No evidence. |
| F2 | 1250-1300 | 0 | Sociopolitical Reorganization: No evidence. |
| Z1 | 1250-1290 | .25 | Pueblo III – Pueblo IV: Increasingly aggregated villages may have allowed for spread of disease but no direct evidence |
| Z2 | 1350-1400 | .25 | Pueblo IV – Protohistoric: aggregated villages may have allowed for spread of disease but no direct evidence |
| S1 | 1275-1325 | 0 | Jacal – Masonry: We have no skeletal evidence from the jacal period and little from the early masonry, but there is no reason to suspect an increase in disease. |
| S2 | 1400-1425 | .25 | Late Pueblo: Greater aggregation and reliance on highly circumscribed water sources may have led to a greater disease load; skeletally; at Gran Quivira health appears to remain stable, however [92,105,120]. |
| H1 | 1070-1100 | .25 | Sedentary – Classic: Larger concentrations. |
| H2 | 1375-1450 | .25 | End of Classic: Larger concentrations. |
| M1 | 950-1000 | .25 | Pithouse – Classic: More people living together in larger pueblo sites so potential for increased risk of diseases, but no skeletal evidence of health problems. |
| M2 | 1130-1150+ | 0 | End of Classic: Smaller sites, no evidence of disease. |
| MV1 | 880-920 | 0 | End of Pueblo I: Smaller sites, no evidence. |
| MV2 | 1240-1290 | .25 | Pueblo III depopulation: More aggregated, but no skeletal evidence of health problems. |

***POWD –* power differences**

***Was there an increase in power differentials, such that some people increasingly have power over others and the “others’” experience a loss of autonomy?***

**Yes -** Evidence of increased differentiation that clearly disadvantages some class of people

**More yes than no -** Evidence of increased differentiation, but consequences not clear

**More no than yes -** Changes might affect some people’s autonomy, but a distinct shift in the power differential not clear.

**No -** No evidence of shifting power differentials.

| **Transfor-mation** | **Dates (CE)** | ***POWD*** | ***Explanation of code for POWD*** |
| --- | --- | --- | --- |
| GE1 | 980-1000 | 0 | Landnám: May have gained more autonomy. |
| GE2 | 1250-1310 | .25 | Recession: Centralization of authority and central functions esp. the church [1]. |
| GE3 | 1400-1450/70 | 0 | No evidence of change in these years. |
| I1 | 870-890 | .75 | Norse Landnám: Some people very disadvantaged [68]. |
| I2 | 950-1000 | .25 | Consolidation: Rearrangement of administration that might have affected autonomy. |
| I3 | 1250-1300 | .25 | Economic and Political Threshold: Rearrangement of administration that might have affected autonomy [5]. |
| F1 | 800-850 | 0 | Norse Landnám: No evidence. |
| F2 | 1250-1300 | .75 | Sociopolitical Reorganization: Sheep letter codifies restrictions on autonomy [121]. |
| Z1 | 1250-1290 | 0 | Pueblo III – Pueblo IV: There is no evidence for changes in power differentials across this transition [8]. |
| Z2 | 1350-1400 | 0 | Pueblo IV – Protohistoric: There is no evidence for changes in power differentials across this transition [8]. |
| S1 | 1275-1325 | .25 | Jacal – Masonry: Little data concerning power; a change in power relations is inferred from the shift from dispersed jacals to tightly aggregated masonry pueblos [12,13]. Panopticon [122]. |
| S2 | 1400-1425 | .25 | Late Pueblo: Again, we have little data concerning power differences but infer with the increasing elaboration of ritual practice and long distance exchange that some members of Salinas Pueblo society had more social power than others [16,123]. |
| H1 | 1070-1100 | 1 | Sedentary – Classic: In Classic period (post 1100) platform mounds have restricted access; differentiation of residence in pithouses vs. compounds [80,124,125]. |
| H2 | 1375-1450 | 1 | End of Classic: In Late Classic (beginning 1300) settlement becomes increasingly primate; platform mounds become more exclusive [124]. |
| M1 | 950-1000 | .25 | Pithouse – Classic: Slightly more evidence of inequality seen in distribution of ritual structures ([126] but not clear if it affected power differentials or autonomy [127]. |
| M2 | 1130-1150+ | 0 | End of Classic: No evidence that the (slight) inequality in the Classic continues. |
| MV1 | 880-920 | .25 | End of Pueblo I: Some, but inconsistent evidence that inequality increased 840-880 [25,128] but doesn’t continue into the final decades. |
| MV2 | 1240-1290 | .75 | Pueblo III depopulation: Concentration of power in some portions of pueblos [76] and a few especially large community centers [28]. |

# **References**

1. Arneborg J. The Norse settlements in Greenland. In: Brink S, Price N, editors. The Viking world. London and New York: Routledge; 2008. pp. 558–597.

2. Arneborg J. The Roman church in Norse Greenland. Acta Archaeo­logica. 1991;61: 142-150.

3. Vésteinsson O, McGovern TH. The peopling of Iceland. Nor Archaeol Rev. 2012;45: 206–218. doi:10.1080/00293652.2012.721792

4. Vésteinsson O. Shopping for Identities: Norse and Christian in the Viking Age North Atlantic. In: Garipzanov I, Bonté R, editors. Conversion and Identity in the Viking Age. Turnhout, Belgium: Brepols; 2014. pp. 75–91.

5. Karlsson G. Iceland’s 1100 years: The history of a marginal society. London: C. Hurst & Co.; 2000.

6. Arge SV, Sveinbjarnardóttir G, Edwards KJ, Buckland PC. Viking and Medieval Settlement in the Faroes: People, Place and Environment. Hum Ecol. 2005;33: 597–620. doi:10.1007/s10745-005-4745-1

7. Mahler DL. The Stratigraphical Cultural Landscape. In: Andersson H, Ersgård L, Svensson E, editors. Outland Use In Preindustrial Europe. Lund, Sweden: Institute of Archaeology, University of Lund; 1998. pp. 49–62.

8. Peeples MA. Connected Communities: Networks, Identities, and Social Change in the Ancient Cibola World. Tucson: University of Arizona Press; 2018.

9. Kintigh KW. Leadership Strategies in Protohistoric Zuni Towns. In: Mills BJ, editor. Alternative Leadership Strategies in the Prehispanic Southwest. Tucson: University of Arizona Press; 2000. pp. 95–116.

10. Peeples MA. Population history of the Zuni region across the Protohistoric transition: migration, gene flow, and social transformation. In: Villalpando E, McGuire RH, editors. Building transnational archaeologies. Tucson: Arizona State Museum and the University of Arizona; 2014. pp. 93–109.

11. Chamberlin M. Evaluating the cultural origins of complexity in the Ancestral Pueblo world. PhD Dissertation, School of Human Evolution and Social Change, Arizona State University. 2008.

12. Chamberlin M. Plazas, performance, and symbolic power in Ancestral Pueblo religion. In: Glowacki DM, Van Keuren S, editors. Religious transformation in the late pre-Hispanic Pueblo world. Tucson: University of Arizona Press; 2011. pp. 130–152.

13. Chamberlin M, Solometo J. Village formation in the Salinas Province, 1000-1400 C.E. In: Spielmann KA, editor. Landscapes of social transformation in the Salinas Province and the Eastern Pueblo world. Tucson: The University of Arizona Press; 2017. pp. 43–68.

14. Rautman A. Population Aggregation, Community Organization, and Plaza-Oriented Pueblos in the American Southwest. J Field Archaeol. 2000;27: 271–284.

15. Rautman A. Constructing Community: The Archaeology of Early Villages in Central New Mexico [Internet]. Tucson: University of Arizona Press; 2014. Available: https://uapress.arizona.edu/book/constructing-community

16. Graves WM. Power, Autonomy, and Inequality in Rio Grande Puebloan Society, A.D. 1300-1672. Unpublished PhD Dissertation, Arizona State University. 2002.

17. Hayes A, Young JN, Warren AH. Excavation of Mound 7: Gran Quivira National Monument, New Mexico [Internet]. 1981. Available: http://archive.org/details/excavationofmoun00haye

18. Abbott DR. Extensive and long-yerm specialization: Hohokam ceramic production in the Phoenix Basin, Arizona. Am Antiq. 2009;74: 531–557. doi:10.1017/S0002731600048745

19. Abbott DR, Smith AM, Gallaga E. Ballcourts and ceramics: The case for Hohokam marketplaces in the Arizona desert. Am Antiq. 2007;72: 461–484.

20. Abbott DR, editor. Centuries of decline during the Hohokam Classic period at Pueblo Grande [Internet]. Tucson: University of Arizona Press; 2003. Available: https://books-google-com.ezproxy1.lib.asu.edu/books/about/Centuries_of_Decline_during_the_Hohokam.html?id=DopkDQAAQBAJ

21. Hill JB, Clark JJ, Doelle WH, Lyons PD. Prehistoric Demography in the Southwest: Migration, Coalescence, and Hohokam Population Decline. Am Antiq. 2004;69: 689–716.

22. Creel D, Anyon R. New Interpretations of Mimbres Public Architecture and Space: Implications for Cultural Change. Am Antiq. 2003;68: 67–92. doi:10.2307/3557033

23. Hegmon M, Nelson MC, Schollmeyer KG. Experiencing social change: Life during the Mimbres Classic transformation. In: Hegmon M, editor. Archaeology of the human experience. Washington D.C.; 2016. pp. 54–73. Available: http://onlinelibrary.wiley.com/doi/10.1111/apaa.12071/abstract

24. LeBlanc SA. The Mimbres People: Ancient Pueblo Painters of the American Southwest. London: Thames and Hudson; 1983.

25. Schachner G. Ritual Control and Transformation in Middle-Range Societies: An Example from the American Southwest. J Anthropol Archaeol. 2001;20: 168–194.

26. Wilshusen RH, Schachner G, Allison JR. Crucible of Pueblos: The Early Pueblo Period in the Northern Southwest [Internet]. Los Angeles, CA: Cotsen Institute of Archaeology at UCLA; 2012. Available: http://www.ioa.ucla.edu/press/crucible-pueblos

27. Arakawa F. Cyclical cultural trajectories: A case study from the Mesa Verde region. J Anthropol Res. 2012;68: 35–69.

28. Glowacki DM. Living and leaving: A social history of regional depopulation in thirteenth-century Mesa Verde. University of Arizona Press; 2015.

29. Ortman SG. Winds from the North: Tewa origins and historical anthropology. Salt Lake City: University of Utah Press; 2012.

30. Madsen CK. Pastoral Settlement, Farming, and hierarchy in Norse Vatnahverfi, South Greenland. Ph.D. Dissertation, Faculty of Humanities, University of Copenhagen. 2014.

31. Vésteinsson O, Church M, Dugmore A, McGovern T, Newton A. Expensive errors or rational choices: the pioneer fringe in Late Viking Age Iceland. Eur J Post-Class Archaeol. 2014;4: 39–68.

32. Kintigh KW. Settlement, Subsistence, and Society in Late Zuni Prehistory. University of Arizona Press; 1985.

33. Spielmann K, Nelson M, Ingram S, Peeples M. Sustainable Small-Scale Agriculture in Semi-Arid Environments. Ecol Soc. 2011;16. doi:10.5751/ES-03814-160126

34. Doelle WH. Tonto Basin demography in a regional perspective. In: Elson MD, Stark MT, Gregory DA, editors. The Roosevelt Community Development Study: New Perspectives on Tonto Basin Prehistory. Tucson: Center for Desert Archaeology; 1995. Available: https://www.researchgate.net/publication/285118486_Tonto_Basin_demography_in_a_regional_perspective

35. Abbott DR, Foster MS. Site structure, chronology, and population. In: Abbott DR, editor. Centuries of decline during the Hohokam Classic period at Pueblo Grande. Tucson: University of Arizona Press; 2003. pp. 24–47. Available: https://books-google-com.ezproxy1.lib.asu.edu/books/about/Centuries_of_Decline_during_the_Hohokam.html?id=DopkDQAAQBAJ

36. Nelson M, Kintigh K, Abbott D, Anderies J. The Cross-scale Interplay between Social and Biophysical Context and the Vulnerability of Irrigation-dependent Societies: Archaeology’s Long-term Perspective. Ecol Soc. 2010;15. doi:10.5751/ES-03389-150331

37. Hegmon M, Peeples MA, Kinzig AP, Kulow S, Meegan CM, Nelson MC. Social Transformation and Its Human Costs in the Prehispanic U.S. Southwest. Am Anthropol. 2008;110: 313–324.

38. Varien MD, Ortman SG, Kohler TA, Glowacki DM, Johnson CD. Historical ecology in the Mesa Verde region: Results from the Village Ecodynamics Project. Am Antiq. 2007; 273–299.

39. Kohler TA, Varien MD, Wright AM, editors. Leaving Mesa Verde: Peril and change in the thirteenth-century Southwest. Tucson: University of Arizona Press; 2010.

40. Price TD, Arneborg J. The Peopling of the North Atlantic: Isotopic Results from Greenland. J N Atl. submitted;

41. Lynnerup N. The Greenland Norse: a biological-anthropological study. Copenhagen: Commission for Scientific Research in Greenland; 1998.

42. Arneborg J. Cultural borders: Reflections on Norse-Eskimo interaction. In: Gilberg R, Gulløv HC, editors. Fifty years of Arctic research Anthropological studies from Greenland to Siberia. Copenhagen, Denmark; 1997. pp. 41–46.

43. Price TD, Gestsdóttir H. The first settlers of Iceland: an isotopic approach to colonisation. Antiquity. 2006;80: 130–144. doi:10.1017/S0003598X00093315

44. Smith JE II, Robertson L, Tawater A, Jameson B, Osburn G. Techado Spring Pueblo: West-Central New Mexico. Dallas: LER & Sons, Publications; 2009.

45. Schachner G. The Decline of Zuni Glaze Ware Production in the Tumultuous Fifteenth Century. In: Habicht-Mauche JA, Eckert SA, Huntley DL, editors. The Social Life of Pots: Glaze Wares and Cultural Dynamics in the Southwest, AD 1250-1680. Tucson: University of Arizona Press; 2006. pp. 124–141.

46. Hill JB, Peeples MA, Huntley DL, Carmack HJ. Spatializing Social Network Analysis in the Late Precontact U.S. Southwest. Adv Archaeol Pract. 2015;3: 63–77. doi:10.7183/2326-3768.3.1.63

47. Hegmon M, Nelson MC, Ruth S. Abandonment, reorganization, and social change: Analyses of pottery and architecture from the Mimbres region of the American Southwest. Am Anthropol. 1998;100: 148–162.

48. Wilshusen RH, Ortman SG. Rethinking the Pueblo I Period in the San Juan Drainage: Aggregation, Migration, and Cultural Diversity. Kiva. 1999;64: 369–400.

49. Wilshusen RH, Ortman SG, Diederichs S, Glowacki DM, Coffey G. Heartland of the Early Pueblos: The Central Mesa Verde Region. Crucible of Pueblos: The Early Pueblo Period in the Northern Southwest. Los Angeles, CA: Cotsen Institute of Archaeology at UCLA; 2012. pp. 14–34. Available: http://www.ioa.ucla.edu/press/crucible-pueblos

50. Arneborg J. Greenland and Europe. In: Fitzhugh W, Ward E, editors. Vikings: The North Atlantic saga. Washington D.C.: Smithsonian Institution Press; 2000. pp. 304–318.

51. Sveinbjarnardóttir G. Leirker á Íslandi / Pottery found in excavations in Iceland. Reykjavík: Hid íslenska fornleifafélag; Thjódminjasafn Íslands; 1996.

52. Wylie J. The Faroe Islands: Interpretations of History. Lexington, Kentucky: The University Press of Kentucky; 1987.

53. Hegmon M, Freeman J, Kintigh KW, Nelson MC, Oas S, Peeples MA, et al. Marking and making differences: Representational diversity in the U.S. Southwest. Am Antiq. 2016;81: 253–272. doi:10.7183/0002-7316.81.2.253

54. Graves WM. Social risk and conflict among the plaza Pueblos of the Salinas Province, 1000-1400 C.E. In: Spielmann KA, editor. Landscapes of social transformation in the Salinas Province and the Eastern Pueblo world. Tucson: The University of Arizona Press; 2017. pp. 134–150.

55. Herhahn C, Huntley D. Dynamic Knowledgescapes: Rio Grande and Salinas Glaze Ware Production and Exchange. In: Spielmann KA, editor. Landscapes of social transformation in the Salinas Province and the Eastern Pueblo world. Tucson: The University of Arizona Press; 2017. pp. 203–224.

56. Mobley-Tanaka JL. Rio Grande Glaze Ware and the Construction of Membership Regimes in the Late Prehistoric Southwest. In: Spielmann KA, editor. Landscapes of social transformation in the Salinas Province and the Eastern Pueblo world. Tucson: The University of Arizona Press; 2017. pp. 180–202.

57. Spielmann KA. Ritual influences on the development of Rio Grande Glaze A ceramics. Migration and Reorganization: The Pueblo IV Period in the American Southwest. Tempe, AZ: Arizona State University; 1998. pp. 253–262.

58. Warren AH. A petrographic study of the pottery. Contributions to Gran Quivira Archaeology. Washington D.C.: National Park Service; 1981. pp. 67–73.

59. Crown PL. The Role of Exchange and Interaction in Salt-Gila Basin Hohokam Prehistory. Exploring the Hohokam: Prehistoric Desert Peoples of the American Southwest. Albuquerque, NM: University of New Mexico Press; 1991. pp. 383–416.

60. Doyel DE. Hohokam exchange and interaction. Chaco and hohokam: Prehistoric Regional Systems in the American Southwest. Santa Fe, NM: School of American Research Press; 1991. pp. 225–253.

61. Hegmon M, Nelson MC. In sync, but barely in touch: Relations between the Mimbres region and the Hohokam regional system. Hinterlands and Regional Dynamics in the Ancient Southwest. Tucson, AZ: University of Arizona Press; 2007. pp. 70–96.

62. Hegmon M, Kulow S. Painting as Agency, Style as Structure: Innovations in Mimbres Pottery Designs From Southwest New Mexico. J Archaeol Method Theory. 2005;12: 313–334. doi:10.1007/s10816-005-8451-5

63. Nelson MC, Hegmon M, Kulow S, Schollmeyer KG. Archaeological and ecological perspectives on reorganization: A case study from the Mimbres region of the U.S. Southwest. Am Antiq. 2006;71: 403–432. doi:10.1017/S0002731600039755

64. Schachner G, Huntley DL, Duff AI. Changes in Regional Organization and Mobility in the Zuni Region of the American Southwest during the Pueblo III and IV Periods: Insights from INAA Studies. J Archaeol Sci. 2011;38: 2261–2273.

65. Solometo J, Rautman A, Chamberlin M. Social Risk and Conflict among the Plaza Pueblos of the Salinas Province, 1000-1400 C.E. In: Spielmann KA, editor. Landscapes of social transformation in the Salinas Province and the Eastern Pueblo world. Tucson: The University of Arizona Press; 2017. pp. 69–102.

66. Hegmon M, Nelson MC, Anyon R, Creel D, Leblanc SA, Shafer HJ. Scale and time-space systematics in the post-A.D. 1100 Mimbres region of the North American Southwest. The Kiva. 1999;65: 143–166. doi:10.1080/00231940.1999.11758405

67. Roussell A. Farms and Churches in the Mediaeval Norse Settlements of Greenland. 1941.

68. Vésteinsson O. Ethnicity and class in settlement period Iceland. The Viking Age: Ireland and the West Papers from the Proceedings of the Fifteenth Viking Congress, Cork, 18-27 August 2005. Dublin: Four Courts Press; 2010. pp. 494–510.

69. Vésteinsson O. On farm-mounds. Archaeol Isl. 2010;8: 13–39.

70. Vésteinsson O. Defining the medieval in Icelandic archaeology. Medieval Archaeology in Scandinavia and Beyond History, Trends and Tomorrow. AarhusDublin: Aarhus University Press; 2015. pp. 213–234.

71. Arge SV. The Landnám in the Faroes. Arct Anthropol. 1991;28: 101–120.

72. Doyel DE, editor. The Hohokam Village: Site Structure and Organization. Glenwood Springs, Colorado: Southwestern and Rocky Mountain Division of the American Association for the Advancement of Science; 1987.

73. Craig DB, editor. Rewriting Prehistory in the Hohokam Heartland. Tucson: Center for Desert Archaeology; 2000.

74. Hegmon M, Brady JA, Nelson MC. Variability in Classic Mimbres room suites: Implications for household organization and social differences. In: Powell-Marti VS, Gilman PA, editors. Mimbres Society. Tucson: University of Arizona Press; 2006. pp. 45–65.

75. Gilman PA, Shafer HJ. Mimbres Families and Households. Archaeol Southwest Mag. 2003;17: 5.

76. Lipe WD. Social power in the Central Mesa Verde Region, AD 1150-1290. Seeking the center place: Archaeology and ancient communities in the Mesa Verde region. Salt Lake City: University of Utah Press; 2002. pp. 262–284.

77. Bolender DJ, Steinberg JM, Damiata B. Farmstead relocation at the end of the Viking Age: results of the Skagafjördur archaeological settlement survey. Archaeol Isl. 2011; 77–101.

78. Young GV. From the Vikings to the Reformation: A Chronicle of the Faroe Islands up to 1538. Isle of Man: Shearwater Press; 1979.

79. Doyel DE. The Santan Phase of the Phoenix Basin. The Hohokam Village Revisted. Fort Collins, Colorado: Southwest and Rocky Mountain Division of the American Association for the Advancement of Science; 2000. pp. 221–244.

80. Gregory DA. The Morphology of Platform Mounds and the Structure of Classic Period Hohokam Sites. In: Doyel DE, editor. The Hohokam Village: Site Structure and Organization. Glenwood Springs, CO: American Association for the Advancement of Science; 1987. pp. 183–210.

81. Sires EW Jr. Hohokam Architectural Variability and Site Structure during the Sedentary-Classic Transition. The Hohokam Village. Glenwood Springs, Colorado: Southwest and Rocky Mountain Division of the American Association for the Advancement of Science; 1987. pp. 171–182.

82. Anyon R, LeBlanc SA. The Architectural Evolution of Mogollon-Mimbres Communal Structures. Kiva. 1980;45: 252–277.

83. Nelson MC. Mimbres During the Twelfth Century. Tucson: University of Arizona Press; 1999.

84. Duff AI. Western Pueblo identities: Regional interaction, migration, and transformation. Tucson: University of Arizona Press; 2002.

85. Arneborg J, Lynnerup N, Heinemeier J. Human diet and subsistence patterns in Norse Greenland AD c.980 - AD c.1450: Archaeological interpretation. J N Atl. 2012;3: 119–133.

86. McGovern TH, Perdikaris S, Tinsley C. The economy of landnám. The evidence of zooarchaeology. Approaches to Vínland. Reykjavik; 2001.

87. McGovern TH, Vésteinsson O, Friđriksson A, Church M, Lawson I, Simpson IA, et al. Landscapes of Settlement in Northern Iceland: Historical Ecology of Human Impact and Climate Fluctuation on the Millennial Scale. Am Anthropol. 2007;109: 27–51. doi:10.1525/aa.2007.109.1.27

88. Ogilvie AEJ, Woollett J, Smiarowski K, Arneborg J, troelstra S, Kuijpers A, et al. Seals and sea ice in Medieval Greenland. J N Atl. 2009;2: 60–80.

89. Rautman A. Final Report on the Excavation of LA-9032, Frank’s Pueblo, Socorro County, New Mexico. Santa Fe, NM; 2013.

90. Spielmann KA. Colonists, Hunters, and Farmers: Plains-Pueblo Interaction in the 17th Century. In: Thomas DH, editor. Columbian Consequences. Washington, D.C.: Smithsonian Institution Press; 1989. pp. 101–113.

91. Spielmann KA, Angstadt-Leto EA. Hunting, Gathering, and Health in the Prehistoric Southwest. Santa Fe Institute Studies in the Sciences of Complexity. Reading, Massachusetts: Addison-Wesley Publishing Company; 1996. pp. 79–106.

92. Spielmann KA, Clark T, Hawkey D, Rainey K, Fish SK. “…being weary, they had rebelled”: Pueblo subsistence and labor under Spanish colonialism. J Anthropol Archaeol. 2009;28: 102–125. doi:10.1016/j.jaa.2008.10.002

93. Dean RM. Hunting intensification and the Hohokam “collapse.” J Anthropol Archaeol. 2007;26: 109–132. doi:10.1016/j.jaa.2006.03.010

94. Szuter CR. Hunting by Hohokam Desert Farmers. KIVA. 1991;56: 277–291. doi:10.1080/00231940.1991.11758172

95. McClelland JA. Revisiting Hohokam Paleodemography. Am Antiq. 2015;80: 492–510. doi:10.7183/0002-7316.80.3.492

96. Meegan CM. Nutritional stress and the depopulation of the Lower Salt River Valley Hohokam. PhD Dissertation, School of Human Evolution and Social Change, Arizona State University. 2009.

97. Sheridan SG. Childhood health as an indicator of biological stress. In: Abbott DR, editor. Centuries of Decline during the Hohokam Classic Period at Pueblo Grande. Tucson, AZ: University of Arizona Press; 2003. pp. 82–106. Available: https://books-google-com.ezproxy1.lib.asu.edu/books/about/Centuries_of_Decline_during_the_Hohokam.html?id=DopkDQAAQBAJ

98. Schollmeyer KG. Large game, agricultural land, and settlement pattern change in the eastern Mimbres area, southwest New Mexico. J Anthropol Archaeol. 2011;30: 402–415. doi:10.1016/j.jaa.2011.04.004

99. Kuckelman K. The Depopulation of Sand Canyon Pueblo, a Large Ancestral Pueblo Village in Southwestern Colorado. Am Antiq. 2010;75: 497–525. doi:10.7183/0002-7316.75.3.497

100. Bichet V, Gauthier E, Massa C, B. Perren B. Lake Sediments as an Archive of Land use and Environmental Change in the Eastern Settlement, Southwestern Greenland. J N Atl. 2014; 47–63. doi:10.3721/037.002.sp606

101. Dugmore AJ, McGovern TH, Streeter R. Landscape legacies of the Icelandic landnám. What has happened to the environment as a result of settlement, why did it happen, and what have been some of the consequences? Human Ecodynamics in the North Atlantic A Collaborative Model of Humans and Nature through Space and Time. Lanham: Lexington Books; 2014. pp. 195–212.

102. Lawson IT, Edwards KJ, Church MJ, Newton AJ, Cook GT, Gathorne-Hardy FJ, et al. Human impact on an island ecosystem: pollen data from Sandoy, Faroe Islands: Pollen data from Sandoy, Faroe Islands. J Biogeogr. 2008;35: 1130–1152. doi:10.1111/j.1365-2699.2007.01838.x

103. Thomson AM, Simpson IA, Brown JL. Sustainable Rangeland Grazing in Norse Faroe. Hum Ecol. 2005;33: 737–761. doi:10.1007/s10745-005-7596-x

104. Peeples MA, Schachner G, Kintigh KW. The Zuni/Cibola region. The Oxford handbook of Southwest archaeology. Oxford: Oxford University Press; 2017. Available: http://www.oxfordhandbooks.com/view/10.1093/oxfordhb/9780199978427.001.0001/oxfordhb-9780199978427-e-23

105. Strawhacker C, Snitker G, Spielmann KA, Wasiolek M, Sandor J, Kinzig AP, et al. Risk Landscapes and Domesticated Landscapes: Food Security in the Salinas Province. In: Spielmann KA, editor. Landscapes of social transformation in the Salinas Province and the Eastern Pueblo world. Tucson: The University of Arizona Press; 2017. pp. 103–118.

106. Waters MR, Ravesloot JC. Landscape Change and the Cultural Evolution of the Hohokam along the Middle Gila River and Other River Valleys in South-Central Arizona. Am Antiq. 2001;66: 285–299. doi:10.2307/2694609

107. James SR. Hunting and fishing patterns leading to resource depletion. In: Abbott DR, editor. Centuries of Decline during the Hohokam Classic Period at Pueblo Grande. Tucson, AZ: University of Arizona Press; 2003. pp. 70–81. Available: https://books-google-com.ezproxy1.lib.asu.edu/books/about/Centuries_of_Decline_during_the_Hohokam.html?id=DopkDQAAQBAJ

108. Hill JB, Lyons PD, Clark JJ, Doelle WH. The “Collapse” of Cooperative Hohokam Irrigation in the Lower Salt River Valley. J Southwest. 2015;57: 609–674.

109. Minnis PE. Social Adaptation to Food Stress: A Prehistoric Southwestern Example. Chicago: University of Chicago Press; 1985.

110. Sandor JA. Long-term Effects of Prehistoric Agriculture on Soils: Examples from New Mexico and Peru. In: Holliday VT, editor. Soils in Archaeology: Landscape Evolution and Human Occupation. Washington, DC: Smithsonian Institution Press; 1992. pp. 217–245.

111. Hegmon M, Nelson MC, Diehl M, Schollmeyer KG, Elliott M. Agriculture, mobility, and human impact in the Mimbres region of the US Southwest. In: Hantman JJ, Most R, editors. Managing archaeological data and databases: Essays in honor of Sylvia W Gaines. Tempe: Arizona State University; 2006.

112. Kohler TA, Matthews MH. Long-Term Anasazi Land Use and Forest Reduction: A Case Study from Southwest Colorado. Am Antiq. 1988;53: 537–564. doi:10.2307/281216

113. Duff AI, Adams KR, Ryan SC. The impact of long-term residential occupation of community centers on local plant and animal resources. Leaving Mesa Verde: Peril and Change in the Thirteenth-Century Southwest. Tucson, AZ: University of Arizona Press; 2010. pp. 156–179.

114. Arneborg J. The high arctic “utmark” of the Norse Greenlanders: The Norse Greenlander´s use of high arctic resources. In: Andersson H, Ersgård L, Svensson E, editors. Outland use in preindustrial Europe. 1998. pp. 156–167.

115. Howard JB. A paleohydraulic approach to examining agricultural intensification in Hohokam irrigation systems. Res Econ Anthropol. 1993;7: 261–322.

116. Anyon R, Leblanc SA. The Galaz Ruin: A prehistoric Mimbres village in southwestern New Mexico. Albuquerque: Maxwell Museum of Anthropology and the University of New Mexico Press; 1984.

117. Wilcox DR, Robertson G, Wood JS. Organized for War: The Perry Mesa Settlement System and Its Central-Arizona Neighbors. Deadly Landscapes: Case Studies in Prehistoric Southwestern Warfare. Salt Lake City, UT: University of Utah Press; 2001.

118. Potter JM, Chuipka JP. Perimortem mutilation of human remains in an early village in the American Southwest: A case for ethnic violence. J Anthropol Archaeol. 2010;29: 507–523. doi:10.1016/j.jaa.2010.08.001

119. Kohler TA, Ortman SG, Grundtisch KE, Fitzpatrick CM, Cole SM. The better angels of their nature: Declining violence through time among prehispanic farmers of the Pueblo Southwest. Am Antiq. 2014;79: 444–464. doi:10.7183/0002-7316.79.3.444

120. Turner CT II. The Arizona State University Study of Gran Quiviran Physical Anthropology. Contributions to Gran Quivira Archaeology. Washington D.C.: National Park Service; 1981. pp. 119–121.

121. Brewington SD. Tradeoffs and human well-being: Achieving sustainability in the Faroe Islands. In: Hegmon M, editor. Give and take of sustainability: Archaeological and anthropological perspectives on tradeoffs. New York, NY: Cambridge University Press; 2017. pp. 222–243.

122. Graves WM, Keuren SV. Ancestral Pueblo villages and the panoptic gaze of the commune. Camb Archaeol J. 2011;21: 263–282. doi:10.1017/S0959774311000278

123. Graves WM, Spielmann KA. Leadership, Long-Distance Exchange, and Feasting in the Protohistoric Rio Grande. In: Mills BJ, editor. Alternative Leadership Strategies in the Prehispanic Southwest. Tucson: University of Arizona Press; 2000. pp. 45–59.

124. Downum CE, Bostwick TW. The Platform Mound. In: Abbott DR, editor. Centuries of Decline during the Hohokam Classic Period at Pueblo Grande. Tucson, AZ: University of Arizona Press; 2003. pp. 166–200. Available: https://books-google-com.ezproxy1.lib.asu.edu/books/about/Centuries_of_Decline_during_the_Hohokam.html?id=DopkDQAAQBAJ

125. Wilcox DR. Hohokam Social Complexity. Chaco and hohokam: Prehistoric Regional Systems in the American Southwest. Santa Fe, NM: School of American Research Press; 1991. pp. 253–275.

126. Clayton SC. Ritual and Residence: The Social Implications of Classic Mimbres Ceremonial Spaces. Kiva. 2006;72: 71–92.

127. Russell WG. Russell, Will G. 2016 Social Inequality in the Mimbres Region of the U.S. Southwest, ca. 200-1130 C.E. PhD dissertation, SHESC ASU. PhD Dissertation, School of Human Evolution and Social Change, Arizona State University. 2016.

128. Kohler TA, Higgins R. Quantifying Household Inequality in Early Pueblo Villages. Curr Anthropol. 2016;57: 690–697. doi:10.1086/687982
