# Supplementary material for: The human experience of social transformation: Insights from comparative archaeology
Source: PLoS One. 2018 Nov 29;13(11):e0208060. doi: 10.1371/journal.pone.0208060 (PMC6264852; doi:10.1371/journal.pone.0208060)
Supplement: S1 Text — Examples of how the transformation cases were coded. (DOCX) [file pone.0208060.s002.docx]

**S1 Coding Examples.**

Examples demonstrating how the various transformation cases were coded.

1) Landnám means “taking land” in Old Norse, and it is used to refer to the Norse colonization of parts of the North Atlantic, including Greenland, Iceland, and the Faroe Islands, transformation cases **GE1, I1, F1** [1,2]. The colonizers brought material and institutions with them and established new ways of life. In all these cases in-migration was coded as 1 because there was “evidence for the establishment of new communities . . . by immigrants from outside the region.” For these cases, the two key variables institutional breakdown and depopulation were coded as exhibiting no change (INST = 0, DPOP = 0).

2) In the Faroe Islands changes in law and custom were institutionalized with a document known as the “Sheep Letter” in CE 1298, transformation **F2** [3]. Slavery was abolished, but there were new laws prohibiting former slaves from owning land and legitimizing the rights of elite landowners. These changes mostly reinforced existing institutions and there was no depopulation, thus the key variables (INST and DPOP) are coded as 0. Although slavery came to an end, the power of the elite increased, and existing inequalities were codified, thus power differences increased (POWD = .75).

3) Many transformations involve a mix of change and continuity. For example, in the Zuni sequence in the Southwest, the Pueblo III to Pueblo IV (**Z1)** transformation in the late CE 1200s was a rapid shift from dispersed to nucleated settlement with considerable continuity of the population and institutions [4]. Thus, the key variables are coded as 0, but community organization is coded as exhibiting major changes (CORG=1). The later Pueblo IV to Protohistoric (**Z2)** transformation in the late CE 1300s involved some demographic continuity as well as in-migration as people moved to a new series of sites within the region [5,6]. Thus, changes in community organization and in-migration are both coded as having been substantial, but with some continuity (CORG = 0.75, IMG = 0.75).

4) Other transformations involve large population losses or movements and are the termination of a known sequence, including the end of the Norse occupation of Greenland (**GE3),** the end of the Hohokam Classic irrigation system and population decline (**H2),** and the large-scale depopulation of the Mesa Verde region (**MV2).**  In these cases, our analysis focused on the last part of the occupation culminating in the population movement or decline. In all these transformation cases the key variables are coded as exhibiting major changes (INST = 1 and DPOP = 1).

5) The end of the Norse colonies on Greenland (**GE3**) is not well understood and subject to considerable debate. Clearly times were difficult, the colonies were increasingly cut off from trade with Europe (TRD = 1), population decline would have led to a decline in the communal order and thus community security (COMM = 1), and there is evidence of food insecurity and starvation (FDshort = 1), especially in the Western Settlement [7,8]. Some argue that the last settlers died of starvation [7,9] while others think the remaining population left by boat [10]. Thus, the transformation is coded as experiencing institutional breakdown and depopulation (INST = 1, DPOP = 1), but no change in many nature of change variables simply because no change is known.

# References

1. Arge SV. The Landnám in the Faroes. Arct Anthropol. 1991;28: 101–120.

2. Vésteinsson O, McGovern TH. The Peopling of Iceland. Nor Archaeol Rev. 2012;45: 206–218. doi:10.1080/00293652.2012.721792

3. Brewington SD. Tradeoffs and human well-being: Achieving sustainability in the Faroe Islands. In: Hegmon M, editor. Give and take of sustainability: Archaeological and anthropological perspectives on tradeoffs. New York, NY: Cambridge University Press; 2017. pp. 222–243.

4. Peeples MA, Schachner G, Kintigh KW. The Zuni/Cibola region. The Oxford handbook of Southwest archaeology. Oxford: Oxford University Press; 2017. Available: http://www.oxfordhandbooks.com/view/10.1093/oxfordhb/9780199978427.001.0001/oxfordhb-9780199978427-e-23

5. Peeples MA. Population History of the Zuni Region across the Protohistoric Transition: Migration, Gene Flow, and Social Transformation. In: Villalpando E, McGuire RH, editors. Building Transnational Archaeologies. Tucson, AZ: Arizona State Museum and the University of Arizona; 2014. pp. 93–109.

6. Peeples MA. Connected communities: Networks, identities, and social change in the ancient Cibola world. Tucson: University of Arizona Press; 2018.

7. Dugmore AJ, Keller C, McGovern TH. Norse Greenland settlement: Reflections on climate change, trade, and the contrasting fates of human settlements in the North Atlantic islands. Arct Anthropol. 2007;44: 12–36. doi:10.1353/arc.2011.0038

8. Dugmore AJ, McGovern TH, Vésteinsson O, Arneborg J, Streeter R, Keller C. Cultural adaptation, compounding vulnerabilities and conjunctures in Norse Greenland. Proc Natl Acad Sci. 2012;109: 3658–3663. doi:10.1073/pnas.1115292109

9. Dugmore AJ, Keller C, McGovern TH, Casely AF, Smiarowski K. Norse Greenland settlement and limits to adaptation. In: Adger WN, Lorenzoni I, O’Brien K, editors. Adapting to climate change: Thresholds, values, governance. Cambridge: Cambridge University Press; 2009. pp. 96–113.

10. Berglund J. Did the Medieval Norse society in Greenland really fail? In: McAnany PA, Yoffee N, editors. Questioning collapse: human resilience, ecological vulnerability, and the aftermath of empire. Cambridge: Cambridge University Press; 2010. pp. 45–70.
